# Evaluating the effectiveness and utility of a novel culturally-adapted telemonitoring system in improving the glycaemic control of Asians with type-2 diabetes mellitus: a mixed method study protocol

**DOI:** 10.1186/s13063-021-05240-6

**Published:** 2021-04-26

**Authors:** Kuan Liang Shawn Goh, Cia Sin Lee, Choon Huat Gerald Koh, Ng Ling Ling, Seng Bin Ang, Christina Oh, Yongqing Lin, Wei Yuan, Qishi Charles Zheng, Ngiap Chuan Tan

**Affiliations:** 1 SingHealth Polyclinics, Singapore, Singapore; 2 SingHealth-Duke NUS Family Medicine Academic Clinical Programme, Singapore, Singapore; 3 MOH Office of Healthcare Transformation, Singapore, Singapore; 4 Singapore Clinical Research Institute, Singapore, Singapore

**Keywords:** Diabetes, Diabetes mellitus, Telehealth, Telemedicine, Tele-monitoring, Tele-management, Primary care, Culturally adapted, Randomised controlled trial

## Abstract

**Background:**

Regular supervision of patients with type-2 diabetes mellitus (T2DM) by healthcare providers is essential to optimise their glycaemic control but is challenging to achieve in current care models. Telemonitoring is postulated to bridge this gap by leveraging on internet-of-things and mobile-health technology. This study aims to determine the effectiveness of a novel telemonitoring system (OPTIMUM) in improving the glycaemic control of patients with T2DM compared with standard of care alone.

**Methods:**

This mixed-method study comprises an initial randomised controlled trial involving 330 Asian adults with T2DM, aged 26–65 years old with an HbA1c of 7.5–10%, with 115 in the intervention and control arms each. Those in the intervention arm will use standardised Bluetooth-enabled devices to transmit their capillary glucose, blood pressure and weight measurements to the OPTIMUM system. Primary care physicians and nurses will remotely supervise them according to an embedded management algorithm for 6 months, including tele-education via weekly videos over 8 weeks and asynchronous tele-consultation if abnormal or absent parameters are detected. Patients in both arms will be assessed at baseline, 6, 12 and 24 months post-recruitment. The primary outcome will be their HbA1c difference between both arms at baseline and 6 months. Blood pressure and weight control; quality of life, medication adherence, confidence in self-management, diabetic literacy and related distress and healthcare utilisation using validated questionnaires; and incident retinal, renal, cardiac and cerebrovascular complications will be compared between the two arms as secondary outcomes at stipulated time-points. Intervention arm patients will be interviewed using qualitative research methods to understand their experience, acceptance and perceived usefulness of the OPTIMUM system.

**Discussion:**

Overall, this study seeks to evaluate the effectiveness of cultural-adapted telemonitoring system in improving glycaemic control of Asians with type-2 diabetes mellitus compared to standard of care. The results of this trial will better inform policy makers in adopting telemedicine for population health management.

**Trial registration:**

ClinicalTrials.gov NCT04306770. Registered on March 13, 2020.

**Supplementary Information:**

The online version contains supplementary material available at 10.1186/s13063-021-05240-6.

## Background

Worldwide, the number of people living with type-2 diabetes mellitus (T2DM) has quadrupled since 1980 [1]. From 1980 to 2014, the global age-standardised T2DM prevalence has increased from 4.3% to 9.0% [2]. Asia is now the epicentre of the T2DM epidemic and associated cardiovascular complications are the leading cause of morbidity and mortality [3]. In Singapore, the age-adjusted prevalence of T2DM (10.5%) is higher compared to the mean prevalence globally (8.8%) and in South-East Asia (8.5%) [1].

The majority (60%) of patients with TD2M in Singapore are managed at the 18 government primary care clinics or polyclinics due to subsidised fees for consultation, investigations and treatment [4]. T2DM is the third commonest diagnosis in the polyclinic attendances in 2019 [5]. Each polyclinic, staffed by primary care physicians and nurses, provides comprehensive healthcare services, including in-house laboratory, pharmacy, nurse-led retinal and feet screening services to detect T2DM-related complications [4]. Patients with T2DM can walk in or arrange appointments to access the polyclinic services. The current care delivery model comprises on-site clinical and laboratory measurements (such as glycated haemoglobin (HbA1c), lipid and renal panels), counselling and consultation with the polyclinic physicians, nurse clinicians, dieticians and pharmacists in an ambulatory outpatient setting. Aside from a lack of a T2DM register, patients can access multiple healthcare providers. Continuity of care for these patients remains a challenge due to defaulting treatment and a lack of supervision and monitoring in between consultations.

The care delivery system to these patients requires urgent review and enhancement [6], especially with their rising concern to access healthcare services amidst the current COVID-19 pandemic [7]. With the advancement of the internet-of-things, mobile-health, information and communications technology (ICT), their applications in telemedicine can potentially transform the care delivery to these patients [8]. Over the past decade, telemedicine has emerged as a promising solution to bridge the episodic T2DM management.

Telemedicine refers to the systematic provision of healthcare services over separate environments via ICT [9]. In Singapore, it is categorised into tele-collaboration, tele-treatment, telemonitoring and tele-support [9]. A meta-analysis has shown that telemedicine (used interchangeably with telehealth) is effective in improving glycaemic and blood pressure control [10]. However, the selected trials enrolled subjects with baseline HbA1c levels of above 9%, which is skewed towards a subset of high-risk patients and may not be generalisable to others with better glycaemic control. Other limitations include lack of differentiation of the various components of telemedicine and that the study populations were mostly Caucasians. The findings may not be entirely translatable to Asians due to varying levels of acceptance of telemedicine. Sin et al. have shown that only 52% of local patients in polyclinics were willing to use telemonitoring to support their self-management of T2DM and/or hypertension [11]. The construct and design of the telemedicine system need to be patient-centric and culturally-adapted to increase uptake before assessing its outcomes.

A team of primary care professionals have teamed up with a multinational telemedicine vendor to create a telemonitoring system leveraging on the latter’s *VitalHealth* population health platform. Design-thinking, which includes patients’ input, was deployed in developing the telemonitoring system, embedded with educational material, such as locally produced video on T2DM management and evidence-based management algorithm. The system is named OPTIMUM – “*O*ptimising care of *P*atients via *T*elemedicine *I*n *M*onitoring and A*u*gmenting their control of Diabetes *M*ellitus”.

The overarching aim of this mixed-method study is to assess the effectiveness of the OPTIMUM telemonitoring system in improving diabetic management of patients with T2DM by supporting and enhancing their self-management. It includes a randomised control trial (RCT) to compare the glycaemic control, based on the magnitude of HbA1c change at recruitment and at 6th-month post-enrolment, among patients with T2DM in the intervention arm with those in the control arm. The secondary aims include a comparison of blood pressure and weight control; quality of life, medication-adherence, confidence in self-management, diabetic literacy and related distress and healthcare utilisation using validated questionnaires; and incidences of emerging retinal, renal, coronary and cerebrovascular complications of the patients in the two arms at stipulated time-points over 24 months. The study also aims to understand the patients’ experience, acceptance and perceived usefulness of the OPTIMUM system in the intervention arm using qualitative research methods.

## Methods/design

### Study design

**Fig. 1:**
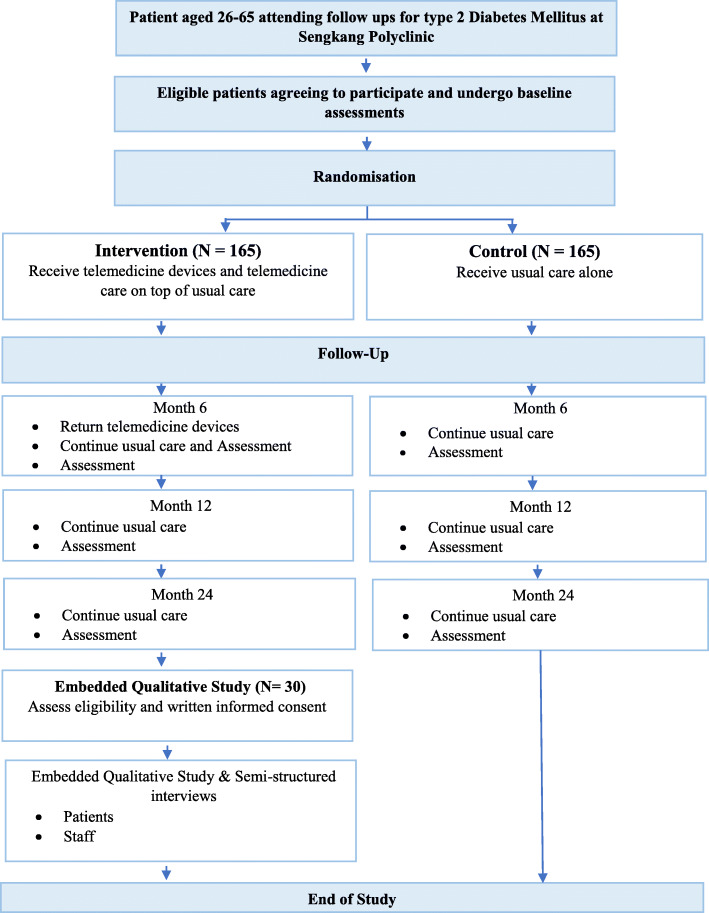
Overview of trial flow

The mixed-method study will begin with an open-label, two-arm, superiority, parallel-group RCT of adult ambulatory patients with T2DM who are managed in primary care. It is followed by qualitative research to gather and understand the perspectives of patients in the intervention arm using in-depth interviews. The study protocol conforms to the Standard Protocol Items Recommendations for Interventional Trials (SPIRIT) checklist and is provided as Additional file 1. The flow diagram to summarise the design of OPTIMUM study is in Fig. 1. The RCT was registered at ClinicalTrials.gov in March 2020 (NCT04306770).

### Setting and participants

The trial will be conducted at a polyclinic located in Sengkang, an estate in north-eastern Singapore with a population of about 300,000 multi-ethnic Asian residents [12]. This polyclinic serves approximately 900 to 1000 patients daily during office hours in each weekday and is similar to the other polyclinics in Singapore [11]. Current standard of care of patients with T2DM at polyclinics include 3–6 monthly doctor’s consultation with HbA1c readings; fasting laboratory panel tests (electrolyte panel, lipid panel, liver panel and urine albumin-creatinine ratio) at diagnosis and then every 6 to 12 months; and annual diabetic retinal photograph and diabetic foot screening. Patients may be referred to dieticians for dietary advice, nurses for co-management or onwards to tertiary care specialists at the discretion of the attending physician.

### Screening

The study team members will pre-screen patients who have the fasting panel tests as described above for eligibility and advise the attending physician to approach them for study enrolment during their medical review a week after the laboratory test.

### Eligibility for study enrolment

Patients will be screened for the following eligibility criteria.

#### Inclusion criteria

1. Diagnosis of T2DM in the patient’s electronic medical record (EMR),
2. Latest glycaemic control based on HbA1c reading between 7% and 10%,
3. Age between 26 and 65 years,
4. No or only mild non-proliferative diabetic retinopathy without any macular involvement,
5. Renal function: estimated glomerular filtration rate (eGFR) of 45 ml/min/1.73 m^2^ or higher (up to stage 3a classification of chronic kidney disease) [13],
6. Non-smokers, or ex-smokers who have quit smoking for at least 12 months,
7. Willingness to download the OPTIMUM smartphone application (app),
8. Willingness to use the standard monitoring devices (blood pressure set, glucometer and weighing scale) which will synchronise with the OPTIMUM telemonitoring system according to the study protocol throughout the 6-month study period,

#### Exclusion criteria

1. Poorly controlled glycaemic control based on the latest HbA1c reading of more than 10%.
2. Cognitive impairment based on a diagnosis of dementia or mild cognitive impairment in the EMR.
3. Pre-existing retinal pathologies documented in the EMR: proliferative diabetic retinopathy (moderate to severe) or other retinal and macular or diseases.
4. Pre-existing chronic kidney disease stage 3b, 4 or 5.
5. Known peripheral vascular, coronary and cerebrovascular disease.
6. Individuals with any end-stage disease with life prognosis of less than 2 years.
7. Pregnant women.
8. Self-declared reluctance or inability to commit to the entire study over 24 months.
9. Self-declared reluctance to use the OPTIMUM smartphone app and the associated monitoring devices.
10. Current enrolment in another trial involving therapeutic or non-therapeutic intervention which will interfere with the primary and secondary outcomes in this study.

### Recruitment

The potential patients who fulfil the eligibility criteria will be provided with an approved patient information sheet by the attending physician during their visit. Their queries and doubts will be addressed by the attending physician before their written endorsement of informed consent is obtained by a clinical research coordinator (CRC). The CRC will then administer standardised questionnaires to record their demographic and clinical data (Table 1) before randomisation. A total of $200 Singapore dollars in grocery vouchers will be given to each participant at recruitment and at each of the study visits to promote participant retention and complete follow-up (Fig. 1).

**Table 1:**
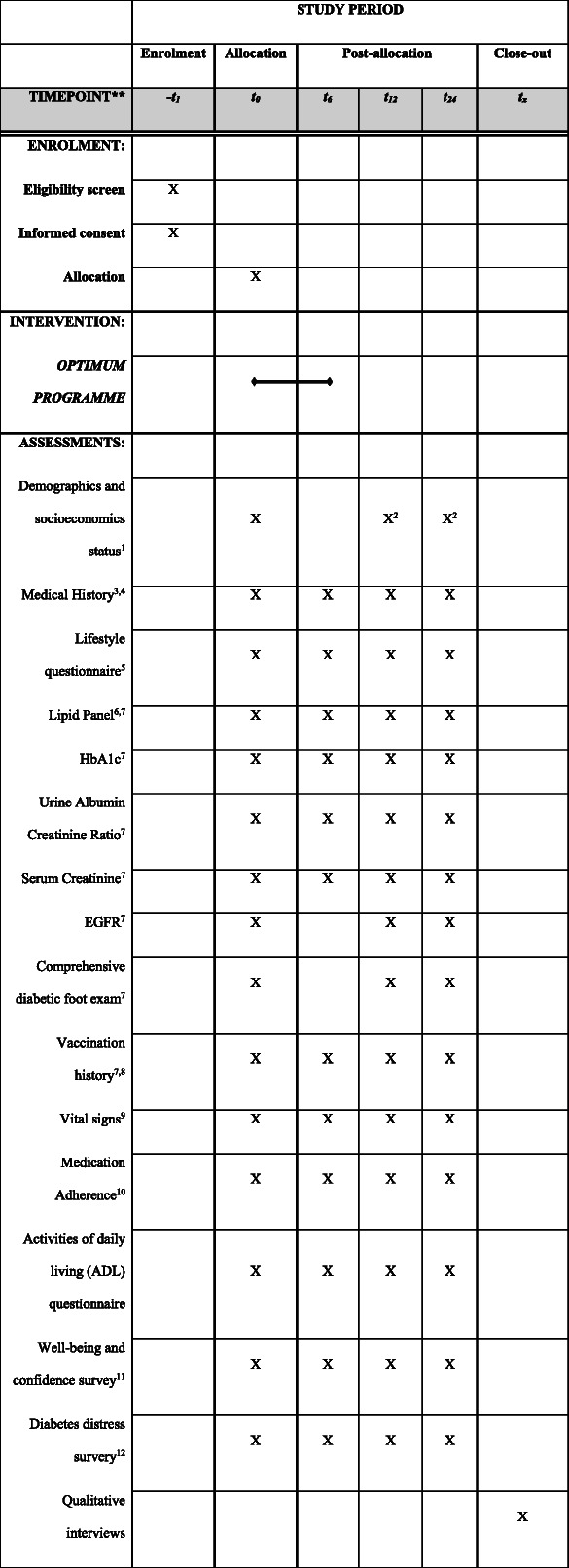
SPIRIT flow diagram of the OPTIMUM trial study protocol

−*t*_1_, baseline assessment; *t*_0_, randomisation (month 0); *t*_6_, postintervention assessments (month 6); *t*_12_, follow up assessments (month 12); *t*_24_, follow-up assessments (month 24); *t*_*x*_, qualitative interviews

1. Demographics includes age, gender, ethnicity. Socioeconomic status includes housing type, employment status, marital status, living status (with children/domestic helper), household monthly income, citizenship and education level.

2. Only employment status will be collected. Other demographics and socioeconomic status will not be collected.

3. Medical history includes diagnosis of hypertension, years of being hypertensive, diagnosis of hyperlipidaemia, years of being hyperlipidaemic, diagnosis of retinopathy, nephropathy, neuropathy, diabetic foot complications, coronary arterial disease, hypoglycaemia, transient ischemic attack or stroke.

4. Years of being diabetic will be included in medical history.

5. Lifestyle survey includes smoking and alcoholic drinking habits and history.

6. Lipid panel includes LDL and HDL cholesterol, triglycerides.

7. All these data will be collected from patients’ medical records 1 month before the recruitment, or within 1 month after the recruitment if the past data is unavailable.

8. History of flu and pneumonia vaccination.

9. Vital signs include systolic and diastolic blood pressure, pulse rate, weight and height.

10. Oral diabetes medication compliance measured using the MARS 5 questionnaire and insulin compliance measured using MPR.

11. Well-being and confidence surveys include Michigan diabetes knowledge, self-care inventory, the international physical activity questionnaire (short) and EQ-5D-5L.

12. Diabetes distress survey using the Problem Areas in Diabetes Scale (PAID).

### Randomisation

Patients will be randomised in a 1:1 ratio to either the intervention or control arm in the RCT. Random permuted blocks will be used to ensure balance over time. The block size will be kept confidential from the study team until the final database lock. The CRCs will refer to the computer-generated block sequence concealed within sealed opaque envelopes to randomise the patients after obtaining their written informed consent (Fig. 1). Each patient will be assigned an individual randomised study identification number after the randomisation. The number assigned will be used to identify the patient and be used for their documentation in the study file.

### Sample size calculation

We assume a clinically significant difference in HbA1c of 0.5% in the sample size calculation based on data published in previous multicentre randomised controlled trials [14, 15]. For a 1:1 allocation ratio and assuming a standard deviation of 1.4 and 1.3 in the intervention and control arms respectively, a sample size of 115 per arm is required to achieve 80% power to detect a difference in HbA1c of 0.5% based on a two-sided 5% significance level. To account for 30% dropout rate over the 24-month follow up, the total sample size was increased to 165 per arm or a total study population of 330 for the RCT. In the subsequent qualitative study, the tentative plan is to interview 30 patients in the intervention group or more until data saturation is reached [16].

### OPTIMUM telemonitoring system

The OPTIMUM system rides on the cloud-based Philips® *VitalHealth* platform for population health management. The platform is currently being used in more than 100 healthcare networks in countries in North America and Europe and will be launched in Singapore in this study. The study team has leveraged on an innovative strategic approach based on Philips® *Co-Create* methodology to culturally adapt the platform to the local Asian population. The *Co-Create* methodology is a step-wise, iterative, people-centric and multi-disciplinary approach to creative innovation and problem-solving. Based on a mindset of collaboration, it extracts thoughts, intentions, and creative ideas via a facilitated workshop. During the workshop, stakeholders, including the researchers, clinicians, nurses, clinic administrators and patients deliberate with the technical team to develop and culturally adapt both the patient-facing smartphone OPTIMUM app and clinician-facing web-app. Two collaborative interviews were conducted through an iterative process to develop and refine the solution.

The OPTIMUM system was developed after iterative solution refinements and two rounds of user acceptance testing. Both the patient OPTIMUM app and clinician web-portal leverage on the Amazon Web Services (AWS)-hosted cloud solution. The cloud-based, containerised microservice architecture makes it a highly accessible and easily scalable health management solution. It also supports role-based authorisation to allow different clinician roles and for them to have access only to specific patient data set to ensure privacy. It is brand- and system-agnostic to various monitoring devices available in the community.

The OPTIMUM system allows for remote patient monitoring, raises their health and diabetes literacy via locally produced patient education videos, and timely interventions via phone call by the clinicians and nurses in the study team when the patients’ clinical parameters exceed pre-determined thresholds. These thresholds are set by the investigators based on local clinical practice guidelines [17] and are embedded in the OPTIMUM management algorithm. The OPTIMUM system facilitates daily monitoring of patients’ parameters and generates tasks based on pre-defined criteria embedded in the OPTIMUM protocol (Additional file 2). The nurses will also take actions according to the protocol algorithm.

### Intervention

A set of three Bluetooth-enabled devices, a weighing scale (Xiaomi Body Composition Scale 2), a glucometer (Accu-chek Guide set, Model 929 mmol/L) and an electronic blood pressure monitor (Smartfuture Medcheck Blood Pressure Monitor SFB01), will be allocated to each patient in the intervention arm for their sole use during the trial. The CRCs or nurses in the study team will induct these patients to the use of the three devices, and synchronise them with the OPTIMUM app. The patients will use the app to provide their responses to the health assessment questionnaires, which will then automatically transmit the data to the nurses for review.

**Fig. 2:**
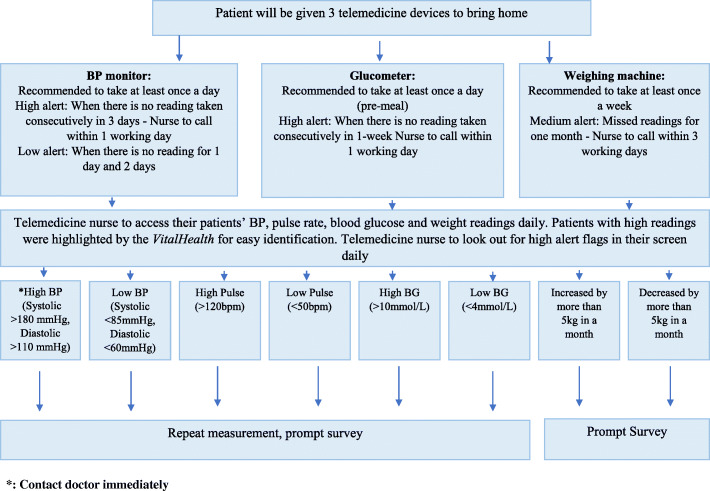
OPTIMUM treatment algorithm

Patients randomised to the control arm will continue with standard care as described previously and may continue using their own devices, if any, for their own monitoring. Patients randomised to the intervention arm are expected to perform the following tasks in the first 6 months after recruitment (Fig. 2).

#### Tele-education

For a period of eight consecutive weeks post-enrolment, patients will receive weekly push reminders for self-monitoring via the OPTIMUM app, links to educational videos covering various aspects of T2DM management (understanding T2DM, eating healthily, alcohol and smoking advice, physical activity demonstrations, and having a healthy mindset). These videos aim to empower patients to improve their self-management of T2DM. After viewing each video, each patient will take a short quiz to test their knowledge related to the video content via the app.

#### Tele-monitoring and tele-support

Patients will self-monitor their body weight, blood pressure, heart rate and capillary glucose levels using the devices according to the stipulated schedule in the OPTIMUM system. The readings will be automatically transmitted through the OPTIMUM system to the clinical care team who will review them asynchronously using the clinician-facing dashboard. Personal target thresholds are pre-set based on clinical practice guidelines (Additional file 2). The study team nurses will phone the patients based on the OPTIMUM treatment algorithm to assess, advise and manage patients accordingly based on their clinical parameters.

Patients will receive immediate feedback via push notifications from the app if their clinical parameters deviate beyond the stipulated range. They will be prompted to respond to questions in the messages to verify their measurements, screen for any related symptoms and assess adherence to medications. The nurses will review their responses before deciding on the appropriate actions based on the pre-specified escalation protocols. The actions include telephone consultations or arranging for patients to return to the polyclinic for assessment in consultation with the clinicians. Patients will also be encouraged to contact the nurses should they have queries about their chronic conditions.

#### Qualitative study procedure

Patients from the intervention arm will be purposively selected from both gender, age and ethnic groups and different level of self-care behaviour (based on their scores in the various scales) to participate in interviews. The one-to-one in-depth interviews will gather qualitative data on their experiences, utility and adherence to self-monitoring using the devices and the OPTIMUM system. Each interview will be audio-recorded, transcribed and audited by an assigned investigator. The topic guide is available as Additional file 3.

### Data

#### Demographic and clinical data

Patients’ sociodemographic and clinical data will be recorded using questionnaires (Table 1). They include age, gender, ethnicity, housing type, employment status, marital status, household information, household monthly income, citizenship and education level; year of diagnoses and duration of T2DM, hypertension, dyslipidaemia, post-enrolment onset of T2DM-related macro, microvascular complications such as coronary, cerebral and peripheral vascular diseases retinopathy, nephropathy, neuropathy, foot complications and hypoglycaemia; adverse events such as hypoglycaemia, hypotension and lifestyle behaviour such as alcoholic intake.

The CRCs will measure their clinical parameters according to the protocol schedule, including systolic and diastolic blood pressure, pulse rate, weight, height and body mass index (BMI).

Demographic data as above will also be collected for participants who drop out and analysed with trial participants for any differences.

#### Laboratory data

Blood investigations are performed at the accredited on-site laboratory or central laboratory at Singapore General Hospital. They include:

1. Glycaemic control: HbA1c (onsite using the DCA® Vantage Analyser).
2. Lipid panel (total cholesterol, HDL-cholesterol, triglycerides and calculated LDL-cholesterol) at central laboratory with the Roche® Cobas c702 machine using the enzymatic colourimetric method.
3. Renal panel (serum electrolytes and creatinine levels) using the Roche® Cobas c702 machine in the central laboratory. The eGFR is computed using the Chronic Kidney Disease Epidemiology Collaboration (CKD-EPI) equation [18].
4. Urine albumin and urine creatinine using the Beckman DXC700AU machine in the central laboratory.

#### T2DM related literacy and behaviour profiles using self-rated scales

1. European Quality of Life-5 dimensions (EQ-5D-5L) scale. It is a brief, generic health-status instrument validated in the local Asian population [19]. The first part of the scale contains 5-point Likert-type items (no/slight/moderate/severe/extreme) to assess the five dimensions of a person’s health status (mobility, self-care, usual activities, pain/discomfort and anxiety/depression). The second part is the EQ-Visual Analog Scale (EQ-VAS), which is a vertical, 0 (the worst health state) to 100 (the best health state) hash-marked numerical scale for self-rating of overall health.
2. Medication adherence is assessed using the Medication Adherence Report Scale (MARS-5) for oral hypoglycaemia agent. The MARS-5 comprises five items which describe a range of non-adherent behaviours, phrased in a non-threatening and non-judgmental way to normalise non-adherence. The scale allows the patients to rate their adherence over five points, rather than on the basis of a “yes/no” or “high/low” dichotomous response, thus providing more detail and differentiation between individuals [20].
3. Medication Possession Ratio (MPR) for insulin. The MPR is a ratio of the total days’ supply of insulin to the number of days of study participation per participant [21].
4. Michigan Diabetes Knowledge Test (MDKT). The MDKT consists of a 14-item general test and a 9-item insulin use subscale to assess literacy on T2DM and treatment [22].
5. Self-care inventory (SCI): Self-care is defined as the daily regimen tasks that a patient performs to manage T2DM. SCI is a self-reported questionnaire to assess patients’ perceptions of self-care behaviours, especially on their adherence to their treatment prescriptions, regardless of their treatment regimens [23].
6. International physical activity questionnaire - short form (IPAQ-SF). The IPAQ-SF records physical activity at four intensity levels: (1) vigorous-intensity activity such as aerobics, (2) moderate-intensity activity such as leisure cycling, (3) walking and (4) sitting [24].
7. Diabetes-related distress using the Problem Area in Diabetes (PAID) scale. It is a 20-item scale to measure emotional distress in people with diabetes mellitus and has been validated in a Singaporean population [25, 26].

### Outcomes

#### Primary outcome measures

The primary outcome will be the mean HbA1c and magnitude of its change on enrolment and at 6 months in patients in the intervention arm compared with those in the control arm.

#### Secondary outcomes at stipulated time points

1. Post-intervention HbA1c at 12 and 24 months.
2. On-site systolic and diastolic blood pressure readings.
3. Weight and BMI.
4. Absolute and change in scores in EQ. 5D-5L, MARS-5, MPR, MDKT, SCI, IPAQ-SF, PAID.
5. Healthcare utilisation based on hospitalisation and polyclinic treatment bills.
6. Incidence of macro and microvascular complications, such as coronary, cerebral, renal retinal and peripheral vascular diseases diagnosed by clinicians and documented in the EMR. Renal outcomes will be determined from the serum creatinine, urine albuminuria and eGFR.
7. Fidelity of patients to the stipulated telemonitoring protocol. It will be computed based on the proportion of them adhering to or exceeding the recommended frequency of self-monitoring of the three key clinical parameters (systolic and diastolic blood pressure, weight and capillary blood glucose) and the proportion of them who have viewed all the health education videos. These data will be retrieved directly from the OPTIMUM system.

### Adverse event reporting

Serious adverse event (SAE) resulting in death, hospitalisation or significant disability and incapacity in patients during the conduct of this study will be immediately reported to the institution review board and documented as SAE data in the eventual analysis of the data.

Adverse event (AE) can be any unfavourable and unintended sign (including an abnormal laboratory finding) and symptoms reported by the patients during the study. AE may be reported directly by patients, via the OPTIMUM app or discovered by the nurses via phone calls. Such information will also be documented and computed for analysis. The Principal Investigator will report the SAE and AE to the Institutional Review Board (CIRB) within the stipulated timeframes.

### Data integrity and governance

The institution and collaborators will adhere to Human Biomedical Research Act guidelines to maintain subject confidentiality. All hard copy research documents will be stored in locked cabinets and electronic datasets will be stored in secured end-to-end encrypted platforms (RedCap and OPTIMUM System) where access is solely restricted to investigators. Quality assurance of the study will be conducted by research auditors from an independent collaborative partner, who will review the study-related source data and documentation. The accuracy of the data will be verified by reviewing all study-related documents. Routine monitoring will be conducted to ensure that the study is conducted in compliance with the currently approved study protocol, standard operating procedures and applicable regulatory requirements.

A Trial Steering Committee comprising of the institution leadership (Executive Director of MOHT and Chief Executive Officer of SingHealth Polyclinics) has been established to review the progress of the study implementation and all reported AE and SAE data every six monthly.

### Statistical analysis

Baseline characteristic (e.g. demographics and socioeconomic status, medical history and lifestyle etc.) will be summarised by arm. The Kolmogorov–Smirnov test will be applied to evaluate the normality of data distribution. For continuous variables, mean and standard deviation will be reported if they are normally distributed, otherwise median and interquartile range will be reported. The number of cases and proportions will be reported for categorical variables.

Continuous outcomes, including mean HbA1c, systolic and diastolic blood pressure, BMI, quality of life, medication adherence and rating scales will be compared by arms. Mean difference with 95% confidence interval will be calculated and tested by two sample *t* test if they are normally distributed, and the non-parametric test will be applied otherwise. The number and proportion of patients experiencing a cerebrovascular event will be tabulated by arm. Difference in proportions will be tested using Fisher’s exact test with 95% confidence interval calculated. One interim analysis with the same abovementioned analyses is planned for this trial when all the patients complete month 6’s follow-up visit. The level of significance will be set at 5%. Data will be analysed using Statistical Analysis System v.9.4.

#### Safety data analyses

The number and proportion of patients experiencing T2DM related complications will be tabulated for each arm with 95% confidence intervals using the exact method. The number and proportion of patients with AE and SAE will be tabulated and compared between both arms.

#### Qualitative data analysis

The initial two to three transcripts will be coded by two investigators with the aid of *NVivo* software to establish a coding frame, before one of them proceeds to complete the coding for the remaining transcripts. The Health Information Technology Acceptance Model will underpin the coding frame. Transcripts will be analysed using the six steps outlined by Braun and Clarke [16]. The emerging themes will be deliberated by the investigators and then finalised prior to manuscript writing for publication.

## Discussion

Appropriate management of T2DM is crucial in mitigating its associated morbidity and mortality. Current models of care of TD2M patients is episodic and often resource intensive. Gaps in monitoring patient between visits exist and continuity of care is key to optimising glycaemic control.

Telemedicine is promising in how care delivery can be modified to improve glycaemic control through continuous monitoring of patients by leveraging on ICT.

The OPTIMUM programme is a novel multicomponent primary care telemedicine programme comprising of remote patient monitoring, education, and timely interventions utilising a bespoke patient-facing smartphone application and clinician-facing web application. The OPTIMUM *VitalHealth* system is culturally adapted through an iterative and collaborative process in close consultation with clinicians, patients and technical staff.

While previous studies have shown a positive impact of telemedicine in the treatment of T2DM patients, baseline characteristics of these patients were largely poorly controlled. Furthermore, these previous studies were done in a Western population which may not be generalizable to an Asian population. We will thus determine the effectiveness and acceptability of a culturally adapted telemedicine in supporting standard of care of T2DM patients.

Findings from this study will enable provide insight into the acceptability of such a model of T2DM and possibly chronic diseases as a whole. It will also better inform policy makers in adopting telemedicine for population health management.

## Supplementary Information

**Additional file 1.** SPIRIT 2013 Checklist: Recommended items to include in a clinical trial protocol.

**Additional file 2.** OPTIMUM protocol intervention treatment algorithm. (PPTX 139 kb)

**Additional file 3.** Topic guide for the qualitative study.

## Data Availability

Not applicable

## Abbreviations

AE: Adverse event
AWS: Amazon Web Services
BMI: Body mass index
CKD-EPI: Chronic Kidney Disease Epidemiology Collaboration equation
CRC: Clinical research coordinator
EMR: Electronic medical record
eGFR: Estimated glomerular filtration rate
EQ-5D-5L: European Quality of Life-5 dimensions Scale
EQ-VAS: EQ-Visual Analog Scale
ICT: Information and communications technology
IPAQ-SF: International physical activity questionnaire - short form
MARS-5: Medication Adherence Report Scale
MDKT: Michigan Diabetes Knowledge Test
MPR: Medication Possession Ratio
PAID: Diabetes-related distress using the Problem Area in Diabetes
T2DM: Type-2 diabetes mellitus
RCT: Randomised controlled trial
SAE: Serious adverse event
SCI: Self-care inventory

## References

[1] World Health Organization. Global Health Estimates 2015: Disease burden by Cause, Age, Sex, by Country and by Region, 2000-2015. Geneva, 2016.

[2] Worldwide trends in diabetes since 1980: a pooled analysis of 751 population-based studies with 4.4 million participants. Lancet. 2016;387(10027):1513–30. 10.1016/S0140-6736(16)00618-8. 10.1016/S0140-6736(16)00618-8 PMC5081106 27061677

[3] Zheng Y, Ley SH, Hu FB (2018). Global aetiology and epidemiology of type 2 diabetes mellitus and its complications. Nat Rev Endocrinol.

[4] Jafar et al. Management of hypertension and multiple risk factors to enhance cardiovascular health in Singapore: the SingHypertension cluster randomized trial. Trials. 2018;19:180. 10.1186/s13063-018-2559-x PMC5852962 29540213

[5] Top 4 Conditions of Polyclinic Attendances [Internet]. [cited 2020 Jul 22]. Available from: https://www.moh.gov.sg/resources-statistics/singapore-health-facts/top-4-conditions-of-polyclinic-attendances.

[6] Kahn R, Anderson JE (2009). Improving diabetes care: the model for health care reform. Diabetes Care.

[7] Wong JEL, Leo YS, Tan CC (2020). COVID-19 in Singapore - current experience: critical global issues that require attention and action. JAMA.

[8] Muegge BD, Tobin GS (2016). Improving diabetes care with technology and information management. Mo Med.

[9] Ministry of Health, Singapore. National telemedicine guidelines for Singapore. 2015. Available from. https://www.moh.gov.sg/content/moh_web/home/Publications/guidelines.html. [cited 2020 Jul 22].

[10] Wu C, Wu Z, Yang L, et al. Evaluation of the clinical outcomes of telehealth for managing diabetes: A PRISMA-compliant meta-analysis. Medicine. 2018;97(43):e12962–e62. 10.1097/MD.0000000000012962. 10.1097/MD.0000000000012962 PMC6221638 30412116

[11] Sin DYE, et al. Assessment of willingness to Tele-monitoring interventions in patients with type 2 diabetes and/or hypertension in the public primary healthcare setting. BMC Med Inform Decis Mak. 2020;20(1):11. 10.1186/s12911-020-1024-4. 10.1186/s12911-020-1024-4 PMC6986094 31992288

[12] Singapore, D.o.S. Population trends 2019, D.o.S. Singapore, Editor: Department of Statistics Singapore website; 2017. ISSN 2591-8028.

[13] Webster AC, Nagler EV, Morton RL, Masson P (2017). Chronic kidney disease. Lancet.

[14] Paré G, Jaana M, Sicotte C (2007). Systematic review of home Telemonitoring for chronic diseases: the evidence base. J Am Med Inform Assoc.

[15] Wild SH, Hanley J, Lewis SC, McKnight JA, McCloughan LB, Padfield PL, et al. Supported telemonitoring and glycemic control in people with type 2 diabetes: the telescot diabetes pragmatic multicenter randomized controlled trial. PLoS Med. 2016;13(7):e1002098. 10.1371/journal.pmed.1002098 PMC4961438 27458809

[16] Braun V, Clarke V. Using thematic analysis in psychology. Qual Res Psychol. 2006;3:77–101. 10.1191/1478088706qp063oa.

[17] Ministry of Health, Singapore. Clinical Practice Guidelines (Medical). [Internet]. [cited 2020 Jul 22]. Available from. https://www.moh.gov.sg/hpp/all-healthcare-professionals/guidelines/GuidelineDetails/clinical-practice-guidelines-medical.

[18] Levey AS, Bosch JP, Lewis JB, Greene T, Rogers N, Roth D. A more accurate method to estimate glomerular filtration rate from serum creatinine: a new prediction equation. Ann Intern Med. 1999;130(6):461–70. 10.7326/0003-4819-130-6-199903160-00002 10075613

[19] Wang Y, Tan NC, Tay EG, Thumboo J, Luo N (2015). Cross-cultural measurement equivalence of the 5-level EQ-5D (EQ-5D-5L) in patients with type 2 diabetes mellitus in Singapore. Health Qual Life Outcomes.

[20] Chan AHY, Horne R, Hankins M, Chisari C (2020). The medication adherence report scale: a measurement tool for eliciting patients’ reports of nonadherence. Br J Clin Pharmacol.

[21] Hess LM, Raebel MA, Conner DA, Malone DC (2006). Measurement of adherence in pharmacy administrative databases: a proposal for standard definitions and preferred measures. Ann Pharmacother.

[22] Fitzgerald JT, Funnell MM, Anderson RM, Nwankwo R, Stansfield RB, Piatt GA (2016). Validation of the revised brief diabetes knowledge test (DKT2). Diabetes Educ.

[23] Weinger K, et al. Measuring diabetes self-care: a psychometric analysis of the self-care inventory-revised with adults. Diabetes Care. 2005;28(6):1346–52. 10.2337/diacare.28.6.1346. 10.2337/diacare.28.6.1346 PMC1615849 15920050

[24] Craig CL, Marshall AL, Sjöström M, Bauman A, Booth ML, Ainsworth BE, Pratt M, Ekelund U, Yngve A, Sallis JF, Oja P. International physical activity questionnaire: 12-country reliability and validity. Med Sci Sports Exerc. 2003;35:1381–95. 10.1249/01.MSS.0000078924.61453.FB. 10.1249/01.MSS.0000078924.61453.FB 12900694

[25] Welch GW, Jacobson AM, Polonsky WH (1997). The problem areas in diabetes scale: an evaluation of its clinical utility. Diabetes Care.

[26] Venkataraman K, et al. Psychometric properties of the problem areas in diabetes (PAID) instrument in Singapore. PLoS One. 2015;10(9):e0136759. 10.1371/journal.pone.0136759 PMC4559380 26336088

