# Supplementary material for: Evaluating the effectiveness and utility of a novel culturally-adapted telemonitoring system in improving the glycaemic control of Asians with type-2 diabetes mellitus: a mixed method study protocol
Source: Trials. 2021 Apr 26;22:305. doi: 10.1186/s13063-021-05240-6 (PMC8072297; doi:10.1186/s13063-021-05240-6)
Supplement: Supplementary file 2 — Additional file 2. OPTIMUM protocol intervention treatment algorithm. (PPTX 139 kb) [file 13063_2021_5240_MOESM2_ESM.pptx]

## Slide 1
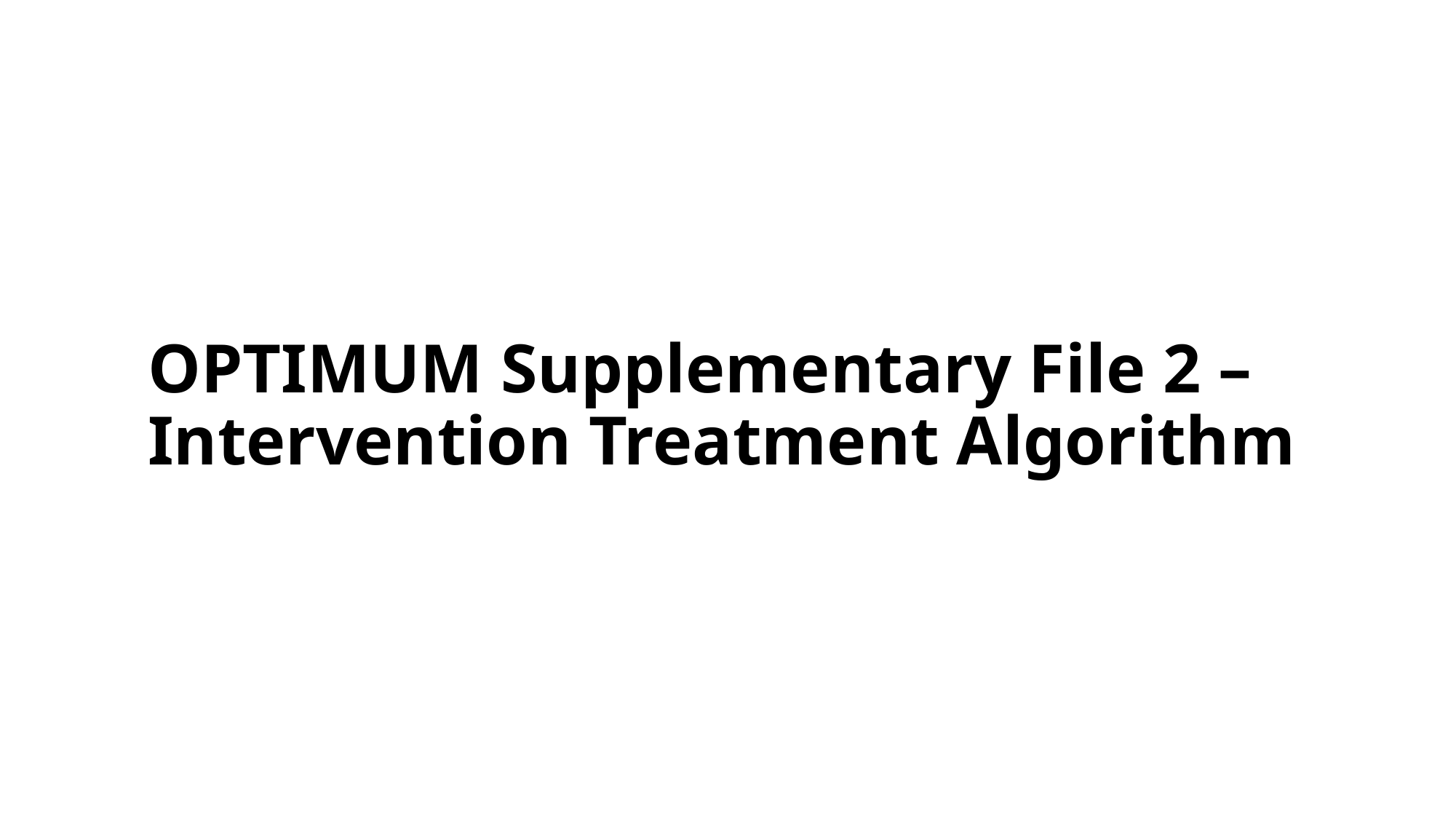

# OPTIMUM Supplementary File 2 – Intervention Treatment Algorithm

## Slide 2
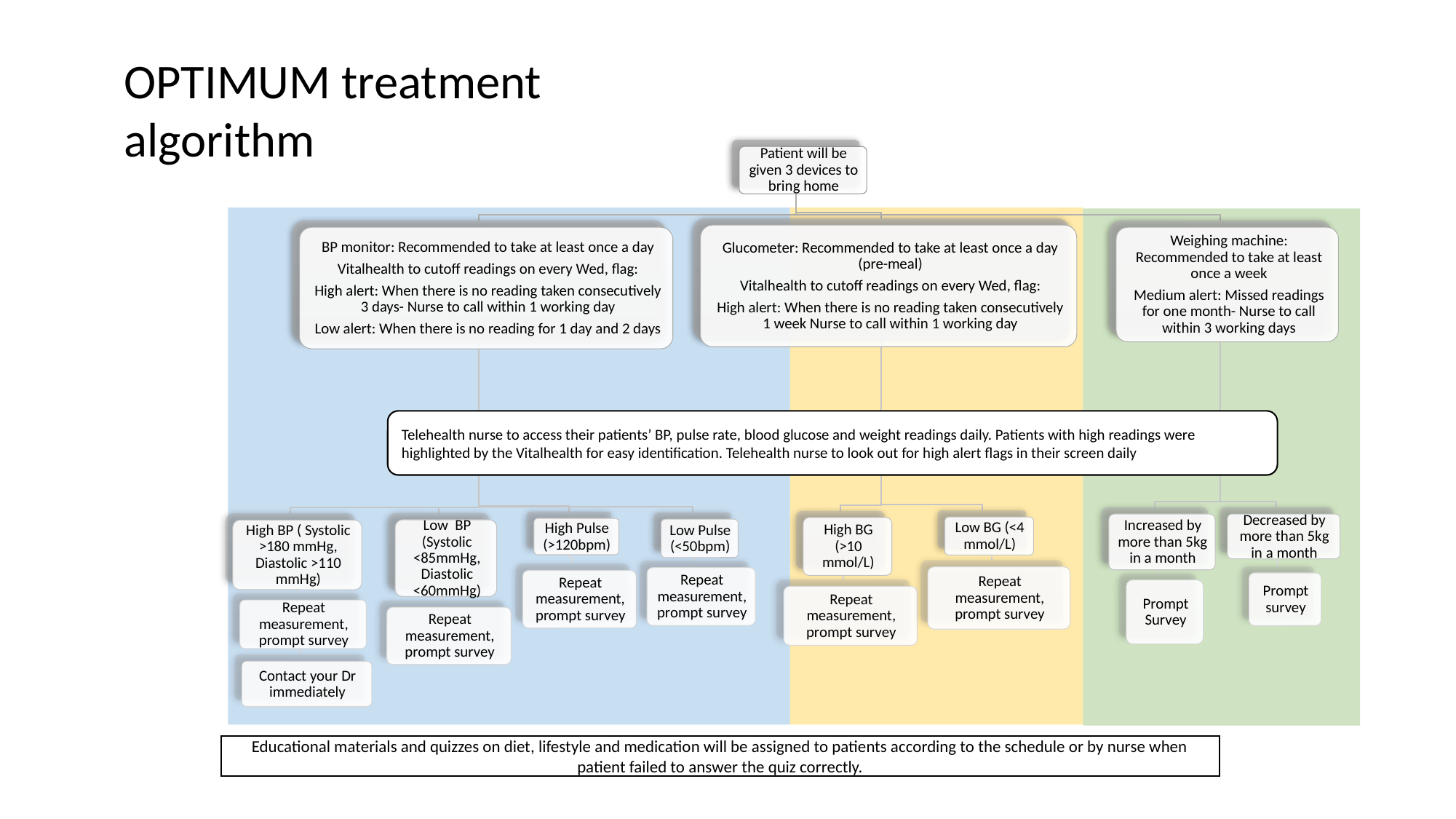

OPTIMUM treatment algorithm
Telehealth nurse to access their patients’ BP, pulse rate, blood glucose and weight readings daily. Patients with high readings were highlighted by the Vitalhealth for easy identification. Telehealth nurse to look out for high alert flags in their screen daily
Educational materials and quizzes on diet, lifestyle and medication will be assigned to patients according to the schedule or by nurse when patient failed to answer the quiz correctly.

## Slide 3
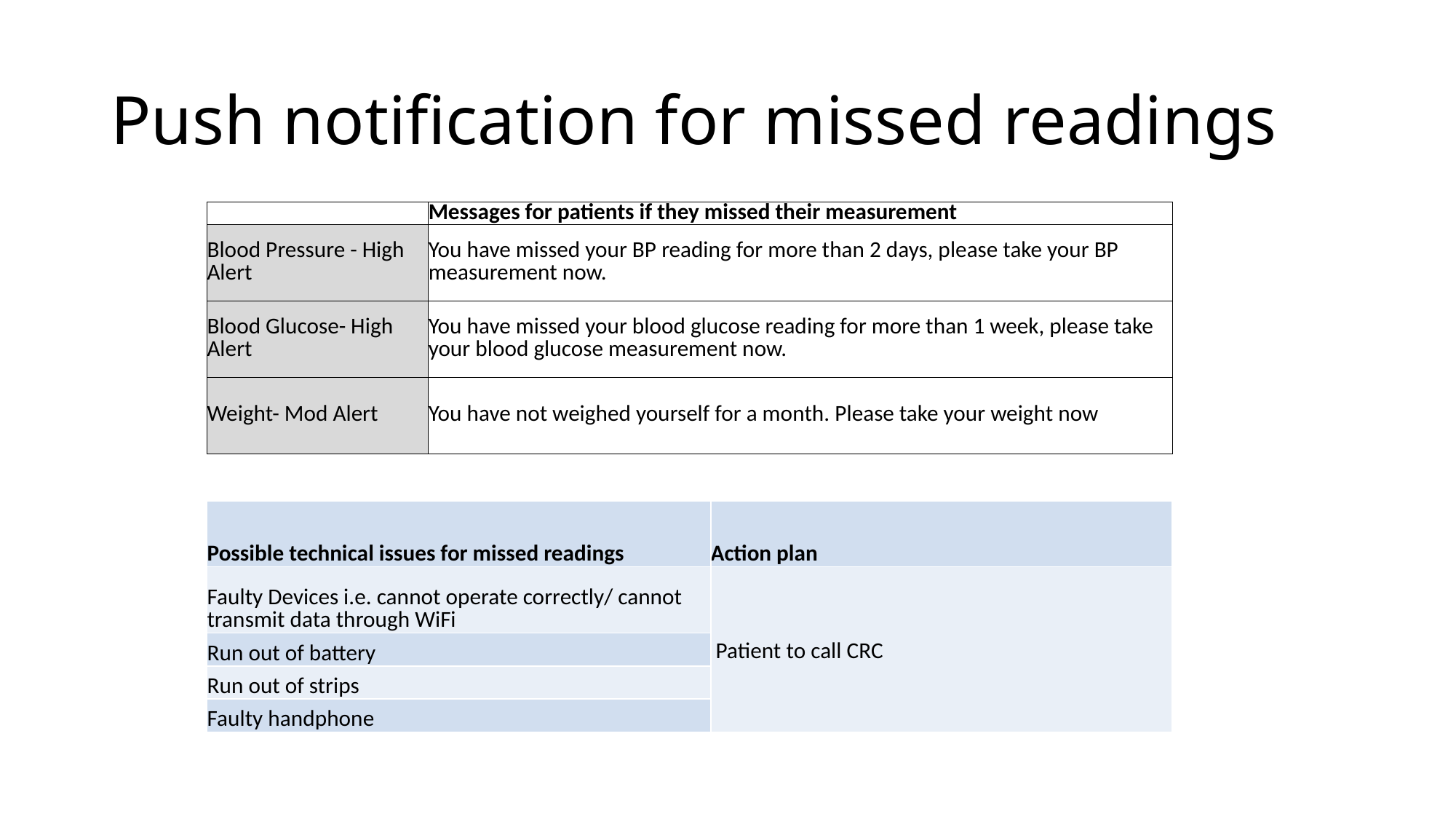

# Push notification for missed readings
| | Messages for patients if they missed their measurement |
| --- | --- |
| Blood Pressure - High Alert | You have missed your BP reading for more than 2 days, please take your BP measurement now. |
| Blood Glucose- High Alert | You have missed your blood glucose reading for more than 1 week, please take your blood glucose measurement now. |
| Weight- Mod Alert | You have not weighed yourself for a month. Please take your weight now |
| Possible technical issues for missed readings | Action plan |
| --- | --- |
| Faulty Devices i.e. cannot operate correctly/ cannot transmit data through WiFi | Patient to call CRC |
| Run out of battery | |
| Run out of strips | |
| Faulty handphone | |

## Slide 4
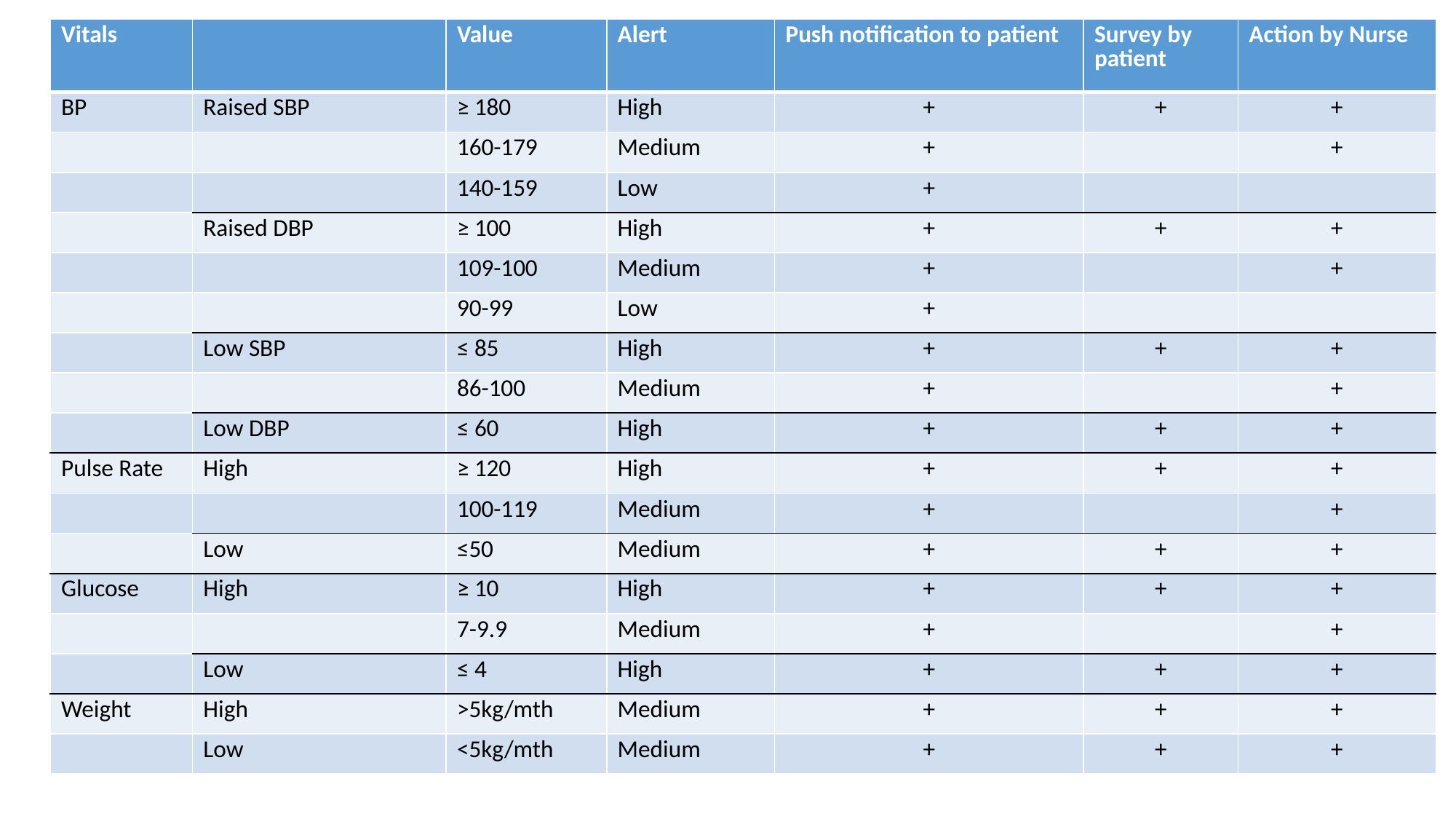

| Vitals | | Value | Alert | Push notification to patient | Survey by patient | Action by Nurse |
| --- | --- | --- | --- | --- | --- | --- |
| BP | Raised SBP | ≥ 180 | High | + | + | + |
| | | 160-179 | Medium | + | | + |
| | | 140-159 | Low | + | | |
| | Raised DBP | ≥ 100 | High | + | + | + |
| | | 109-100 | Medium | + | | + |
| | | 90-99 | Low | + | | |
| | Low SBP | ≤ 85 | High | + | + | + |
| | | 86-100 | Medium | + | | + |
| | Low DBP | ≤ 60 | High | + | + | + |
| Pulse Rate | High | ≥ 120 | High | + | + | + |
| | | 100-119 | Medium | + | | + |
| | Low | ≤50 | Medium | + | + | + |
| Glucose | High | ≥ 10 | High | + | + | + |
| | | 7-9.9 | Medium | + | | + |
| | Low | ≤ 4 | High | + | + | + |
| Weight | High | >5kg/mth | Medium | + | + | + |
| | Low | <5kg/mth | Medium | + | + | + |

## Slide 5
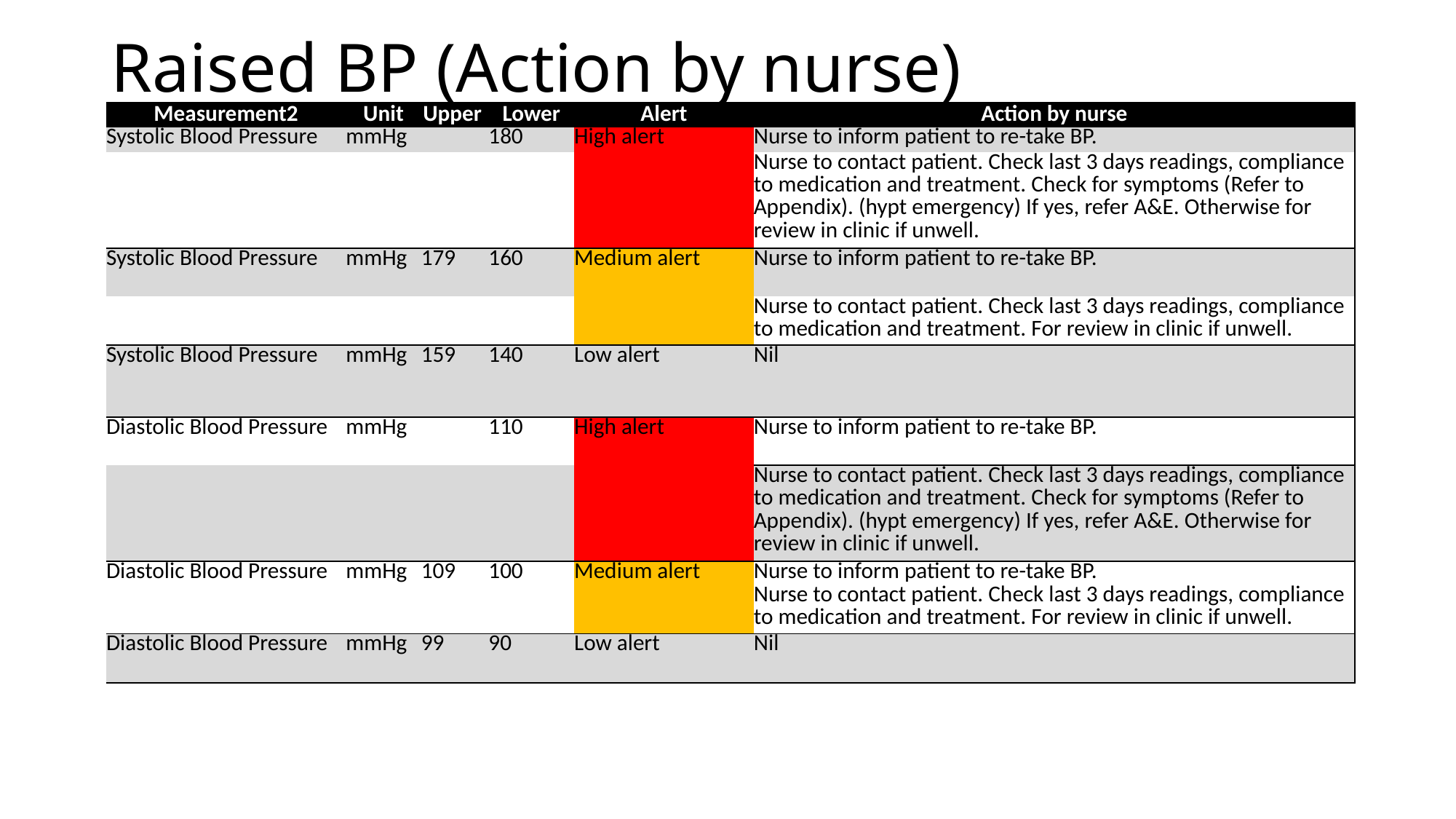

# Raised BP (Action by nurse)
| Measurement2 | Unit | Upper | Lower | Alert | Action by nurse |
| --- | --- | --- | --- | --- | --- |
| Systolic Blood Pressure | mmHg | | 180 | High alert | Nurse to inform patient to re-take BP. |
| | | | | | Nurse to contact patient. Check last 3 days readings, compliance to medication and treatment. Check for symptoms (Refer to Appendix). (hypt emergency) If yes, refer A&E. Otherwise for review in clinic if unwell. |
| Systolic Blood Pressure | mmHg | 179 | 160 | Medium alert | Nurse to inform patient to re-take BP. |
| | | | | | Nurse to contact patient. Check last 3 days readings, compliance to medication and treatment. For review in clinic if unwell. |
| Systolic Blood Pressure | mmHg | 159 | 140 | Low alert | Nil |
| Diastolic Blood Pressure | mmHg | | 110 | High alert | Nurse to inform patient to re-take BP. |
| | | | | | Nurse to contact patient. Check last 3 days readings, compliance to medication and treatment. Check for symptoms (Refer to Appendix). (hypt emergency) If yes, refer A&E. Otherwise for review in clinic if unwell. |
| Diastolic Blood Pressure | mmHg | 109 | 100 | Medium alert | Nurse to inform patient to re-take BP. Nurse to contact patient. Check last 3 days readings, compliance to medication and treatment. For review in clinic if unwell. |
| Diastolic Blood Pressure | mmHg | 99 | 90 | Low alert | Nil |

## Slide 6
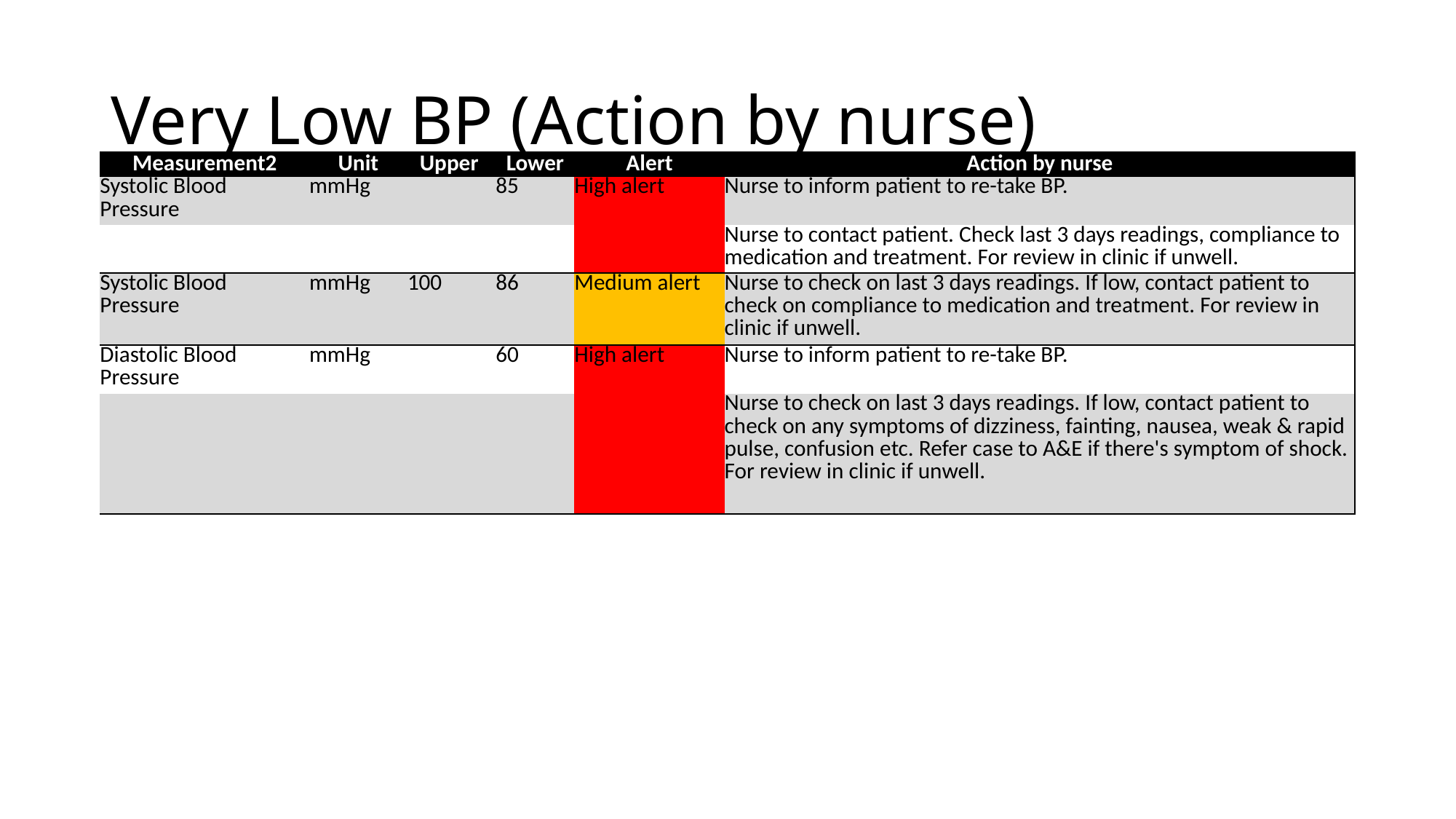

# Very Low BP (Action by nurse)
| Measurement2 | Unit | Upper | Lower | Alert | Action by nurse |
| --- | --- | --- | --- | --- | --- |
| Systolic Blood Pressure | mmHg | | 85 | High alert | Nurse to inform patient to re-take BP. |
| | | | | | Nurse to contact patient. Check last 3 days readings, compliance to medication and treatment. For review in clinic if unwell. |
| Systolic Blood Pressure | mmHg | 100 | 86 | Medium alert | Nurse to check on last 3 days readings. If low, contact patient to check on compliance to medication and treatment. For review in clinic if unwell. |
| Diastolic Blood Pressure | mmHg | | 60 | High alert | Nurse to inform patient to re-take BP. |
| | | | | | Nurse to check on last 3 days readings. If low, contact patient to check on any symptoms of dizziness, fainting, nausea, weak & rapid pulse, confusion etc. Refer case to A&E if there's symptom of shock. For review in clinic if unwell. |

## Slide 7
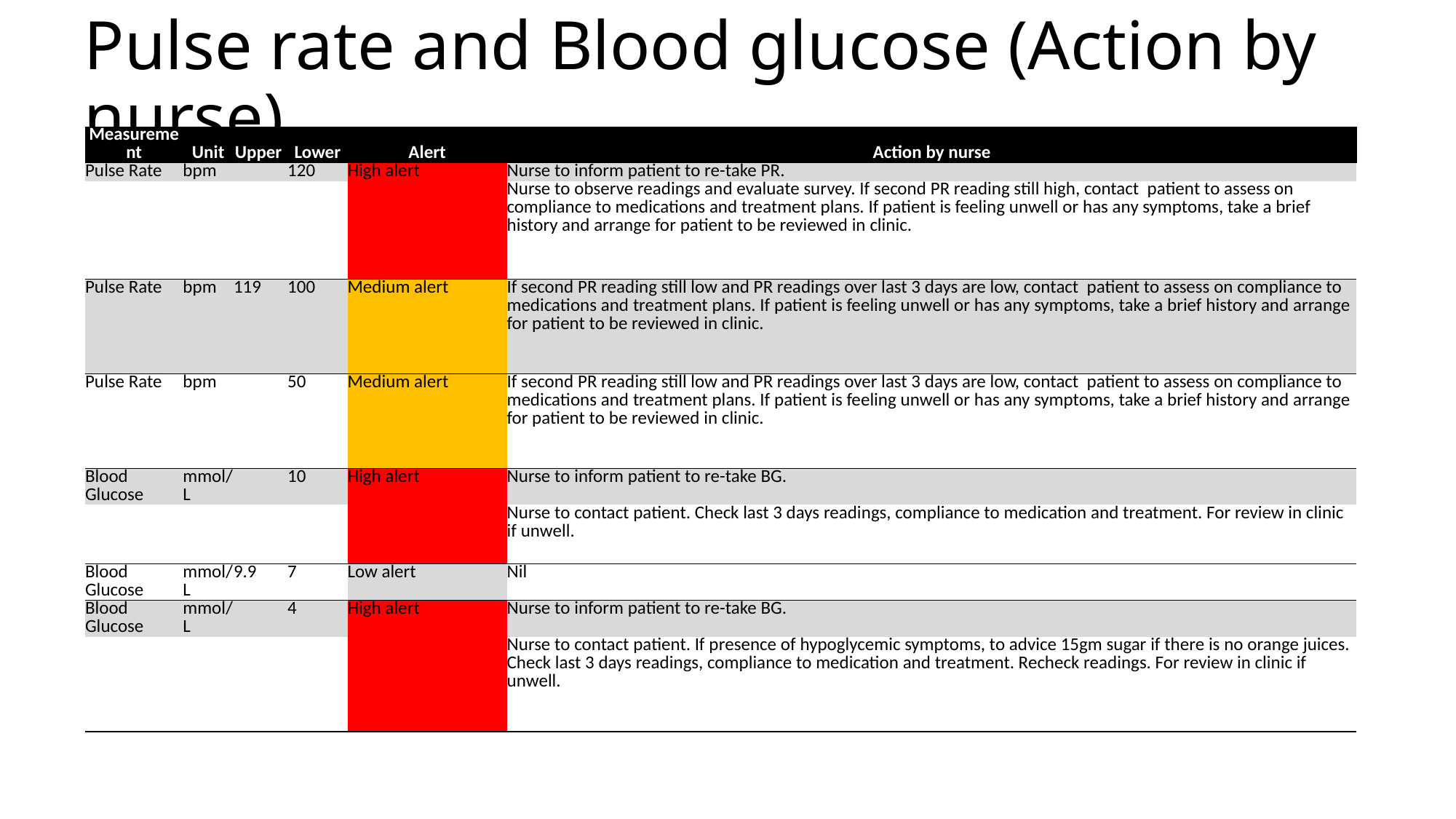

# Pulse rate and Blood glucose (Action by nurse)
| Measurement | Unit | Upper | Lower | Alert | Action by nurse |
| --- | --- | --- | --- | --- | --- |
| Pulse Rate | bpm | | 120 | High alert | Nurse to inform patient to re-take PR. |
| | | | | | Nurse to observe readings and evaluate survey. If second PR reading still high, contact patient to assess on compliance to medications and treatment plans. If patient is feeling unwell or has any symptoms, take a brief history and arrange for patient to be reviewed in clinic. |
| Pulse Rate | bpm | 119 | 100 | Medium alert | If second PR reading still low and PR readings over last 3 days are low, contact patient to assess on compliance to medications and treatment plans. If patient is feeling unwell or has any symptoms, take a brief history and arrange for patient to be reviewed in clinic. |
| Pulse Rate | bpm | | 50 | Medium alert | If second PR reading still low and PR readings over last 3 days are low, contact patient to assess on compliance to medications and treatment plans. If patient is feeling unwell or has any symptoms, take a brief history and arrange for patient to be reviewed in clinic. |
| Blood Glucose | mmol/L | | 10 | High alert | Nurse to inform patient to re-take BG. |
| | | | | | Nurse to contact patient. Check last 3 days readings, compliance to medication and treatment. For review in clinic if unwell. |
| Blood Glucose | mmol/L | 9.9 | 7 | Low alert | Nil |
| Blood Glucose | mmol/L | | 4 | High alert | Nurse to inform patient to re-take BG. |
| | | | | | Nurse to contact patient. If presence of hypoglycemic symptoms, to advice 15gm sugar if there is no orange juices. Check last 3 days readings, compliance to medication and treatment. Recheck readings. For review in clinic if unwell. |

## Slide 8
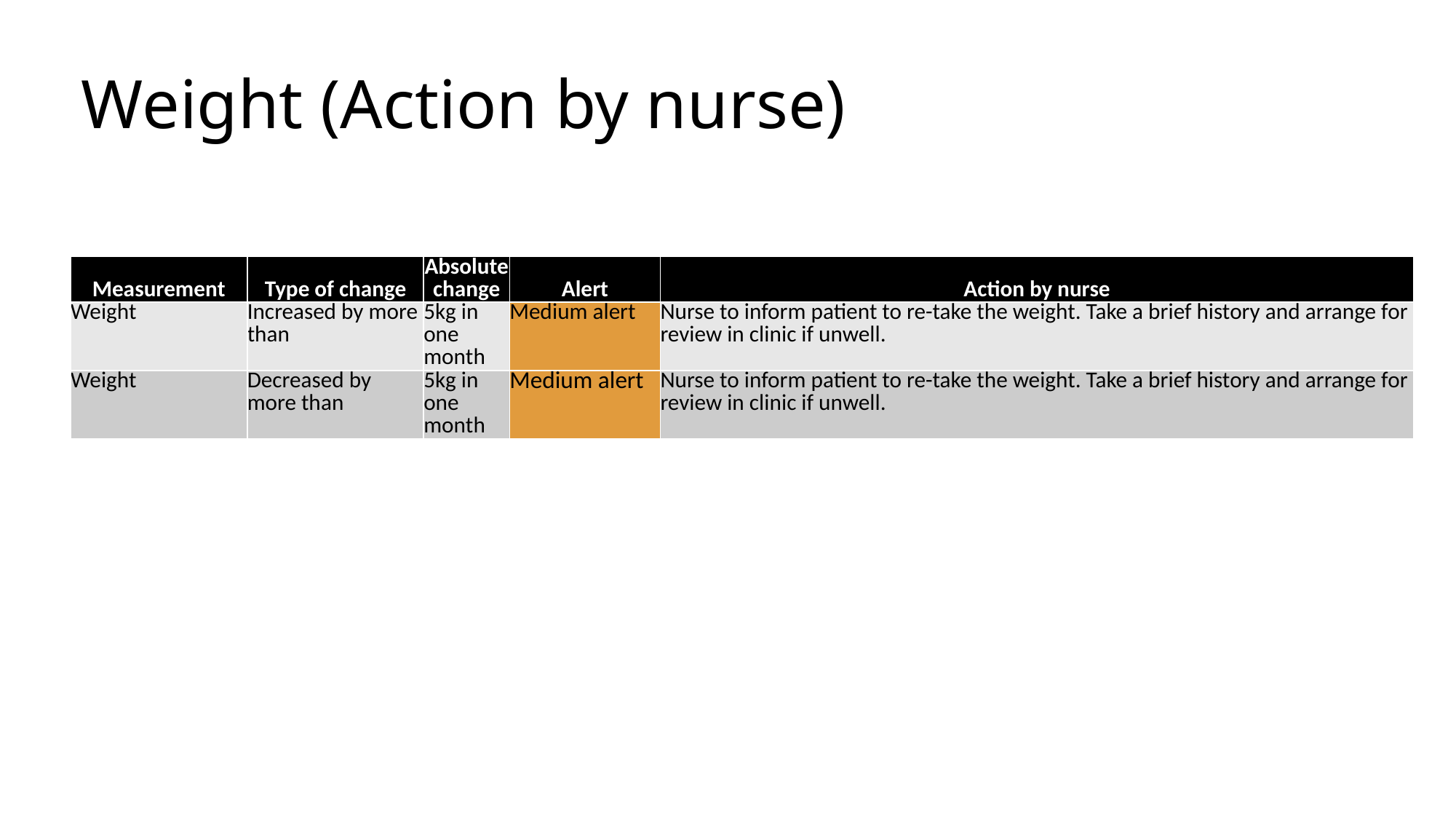

# Weight (Action by nurse)
| Measurement | Type of change | Absolute change | Alert | Action by nurse |
| --- | --- | --- | --- | --- |
| Weight | Increased by more than | 5kg in one month | Medium alert | Nurse to inform patient to re-take the weight. Take a brief history and arrange for review in clinic if unwell. |
| Weight | Decreased by more than | 5kg in one month | Medium alert | Nurse to inform patient to re-take the weight. Take a brief history and arrange for review in clinic if unwell. |

## Slide 9
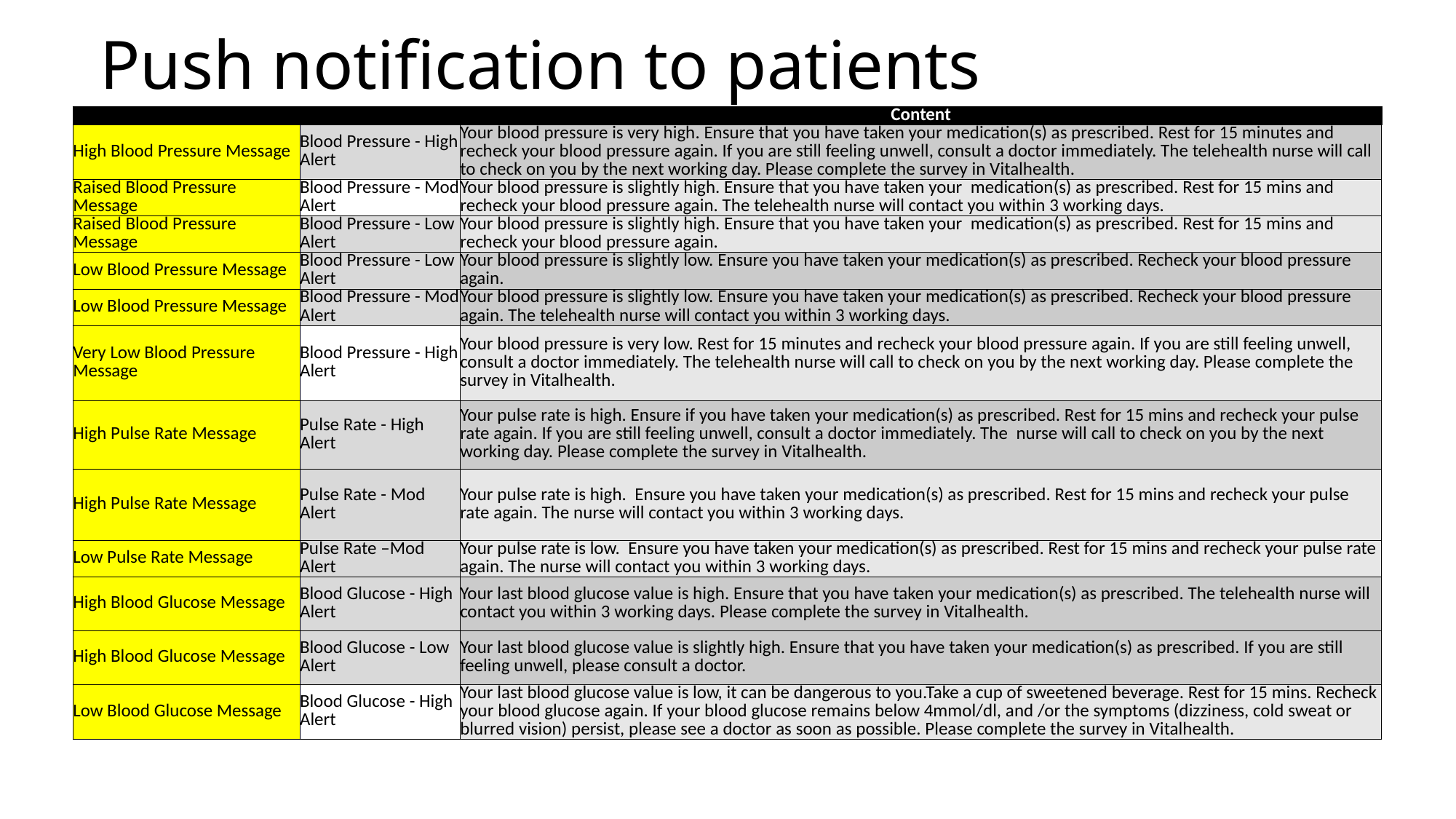

# Push notification to patients
| Name | Schedule | Content |
| --- | --- | --- |
| High Blood Pressure Message | Blood Pressure - High Alert | Your blood pressure is very high. Ensure that you have taken your medication(s) as prescribed. Rest for 15 minutes and recheck your blood pressure again. If you are still feeling unwell, consult a doctor immediately. The telehealth nurse will call to check on you by the next working day. Please complete the survey in Vitalhealth. |
| Raised Blood Pressure Message | Blood Pressure - Mod Alert | Your blood pressure is slightly high. Ensure that you have taken your medication(s) as prescribed. Rest for 15 mins and recheck your blood pressure again. The telehealth nurse will contact you within 3 working days. |
| Raised Blood Pressure Message | Blood Pressure - Low Alert | Your blood pressure is slightly high. Ensure that you have taken your medication(s) as prescribed. Rest for 15 mins and recheck your blood pressure again. |
| Low Blood Pressure Message | Blood Pressure - Low Alert | Your blood pressure is slightly low. Ensure you have taken your medication(s) as prescribed. Recheck your blood pressure again. |
| Low Blood Pressure Message | Blood Pressure - Mod Alert | Your blood pressure is slightly low. Ensure you have taken your medication(s) as prescribed. Recheck your blood pressure again. The telehealth nurse will contact you within 3 working days. |
| Very Low Blood Pressure Message | Blood Pressure - High Alert | Your blood pressure is very low. Rest for 15 minutes and recheck your blood pressure again. If you are still feeling unwell, consult a doctor immediately. The telehealth nurse will call to check on you by the next working day. Please complete the survey in Vitalhealth. |
| High Pulse Rate Message | Pulse Rate - High Alert | Your pulse rate is high. Ensure if you have taken your medication(s) as prescribed. Rest for 15 mins and recheck your pulse rate again. If you are still feeling unwell, consult a doctor immediately. The nurse will call to check on you by the next working day. Please complete the survey in Vitalhealth. |
| High Pulse Rate Message | Pulse Rate - Mod Alert | Your pulse rate is high. Ensure you have taken your medication(s) as prescribed. Rest for 15 mins and recheck your pulse rate again. The nurse will contact you within 3 working days. |
| Low Pulse Rate Message | Pulse Rate –Mod Alert | Your pulse rate is low. Ensure you have taken your medication(s) as prescribed. Rest for 15 mins and recheck your pulse rate again. The nurse will contact you within 3 working days. |
| High Blood Glucose Message | Blood Glucose - High Alert | Your last blood glucose value is high. Ensure that you have taken your medication(s) as prescribed. The telehealth nurse will contact you within 3 working days. Please complete the survey in Vitalhealth. |
| High Blood Glucose Message | Blood Glucose - Low Alert | Your last blood glucose value is slightly high. Ensure that you have taken your medication(s) as prescribed. If you are still feeling unwell, please consult a doctor. |
| Low Blood Glucose Message | Blood Glucose - High Alert | Your last blood glucose value is low, it can be dangerous to you.Take a cup of sweetened beverage. Rest for 15 mins. Recheck your blood glucose again. If your blood glucose remains below 4mmol/dl, and /or the symptoms (dizziness, cold sweat or blurred vision) persist, please see a doctor as soon as possible. Please complete the survey in Vitalhealth. |

## Slide 10
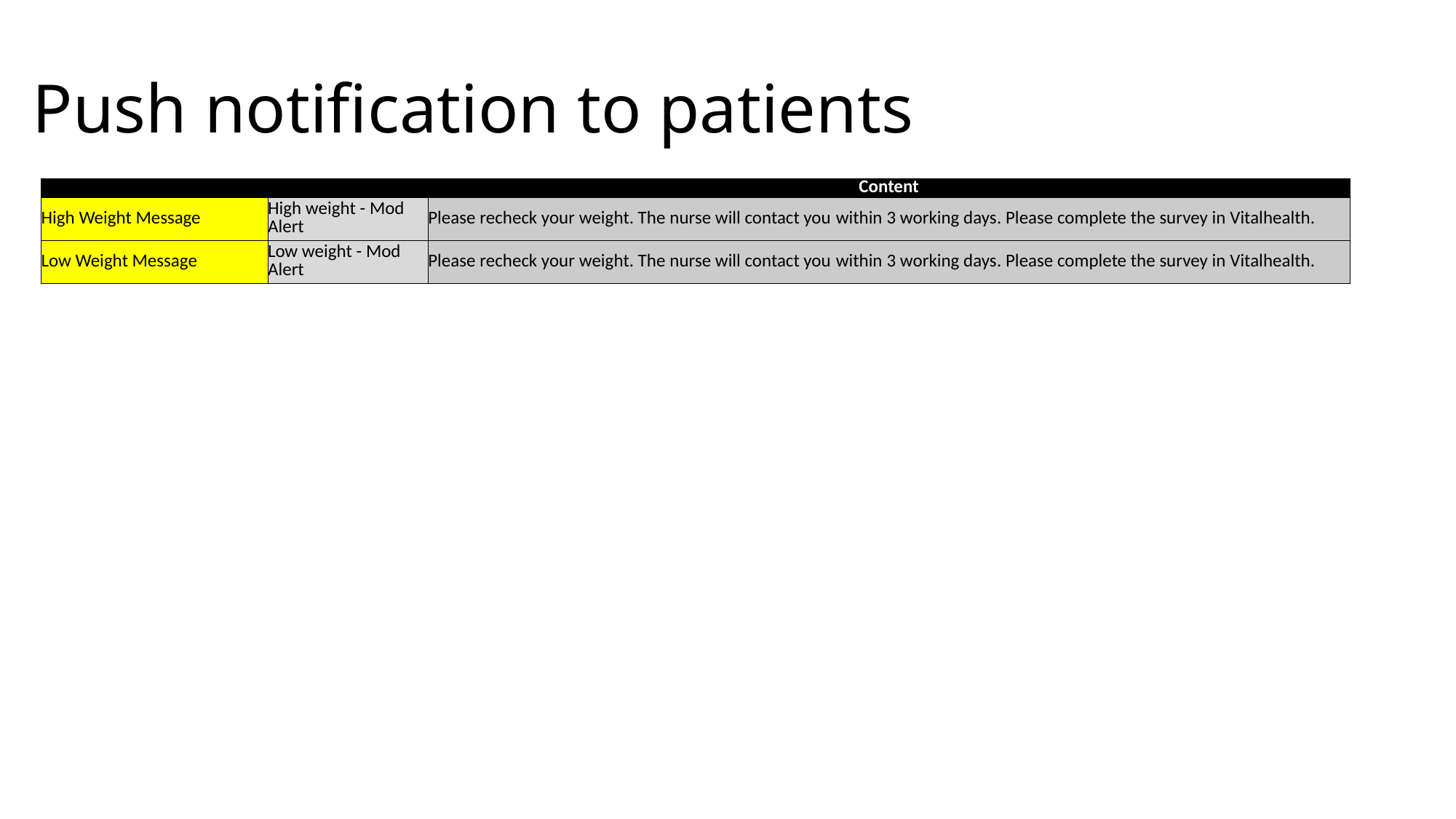

Push notification to patients
| Name | Schedule | Content |
| --- | --- | --- |
| High Weight Message | High weight - Mod Alert | Please recheck your weight. The nurse will contact you within 3 working days. Please complete the survey in Vitalhealth. |
| Low Weight Message | Low weight - Mod Alert | Please recheck your weight. The nurse will contact you within 3 working days. Please complete the survey in Vitalhealth. |

## Slide 11
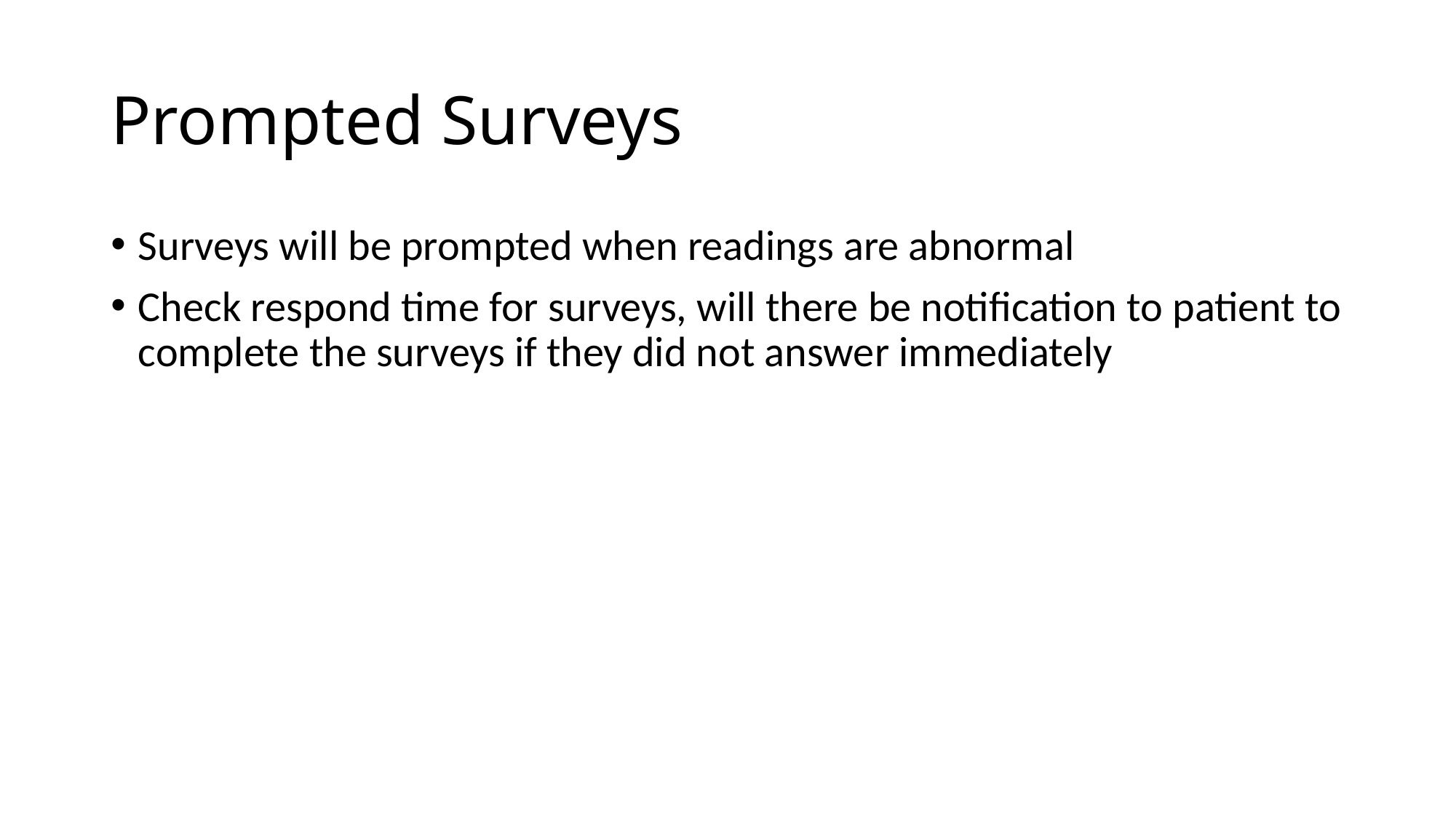

# Prompted Surveys
Surveys will be prompted when readings are abnormal
Check respond time for surveys, will there be notification to patient to complete the surveys if they did not answer immediately

## Slide 12
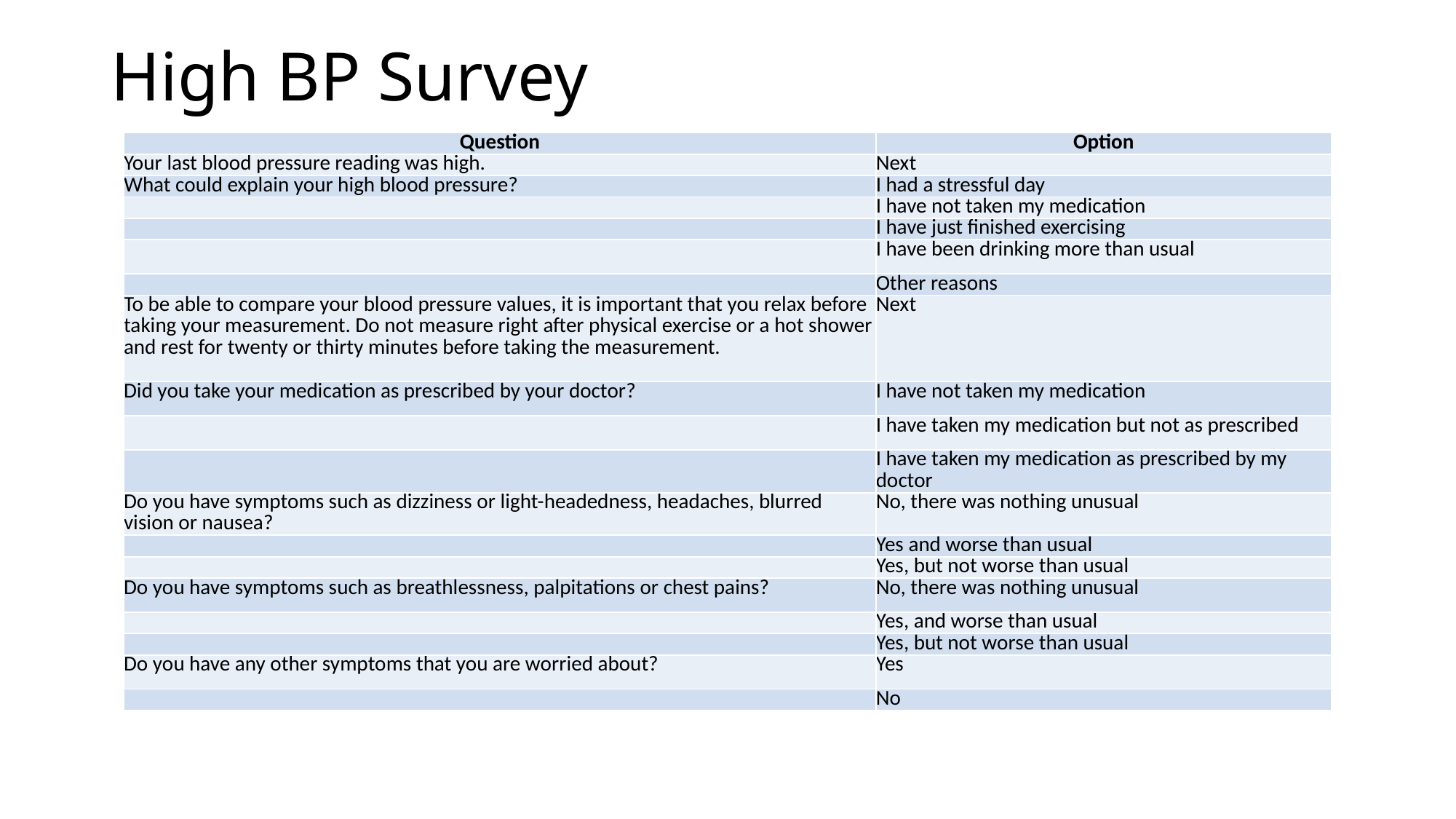

# High BP Survey
| Question | Option |
| --- | --- |
| Your last blood pressure reading was high. | Next |
| What could explain your high blood pressure? | I had a stressful day |
| | I have not taken my medication |
| | I have just finished exercising |
| | I have been drinking more than usual |
| | Other reasons |
| To be able to compare your blood pressure values, it is important that you relax before taking your measurement. Do not measure right after physical exercise or a hot shower and rest for twenty or thirty minutes before taking the measurement. | Next |
| Did you take your medication as prescribed by your doctor? | I have not taken my medication |
| | I have taken my medication but not as prescribed |
| | I have taken my medication as prescribed by my doctor |
| Do you have symptoms such as dizziness or light-headedness, headaches, blurred vision or nausea? | No, there was nothing unusual |
| | Yes and worse than usual |
| | Yes, but not worse than usual |
| Do you have symptoms such as breathlessness, palpitations or chest pains? | No, there was nothing unusual |
| | Yes, and worse than usual |
| | Yes, but not worse than usual |
| Do you have any other symptoms that you are worried about? | Yes |
| | No |

## Slide 13
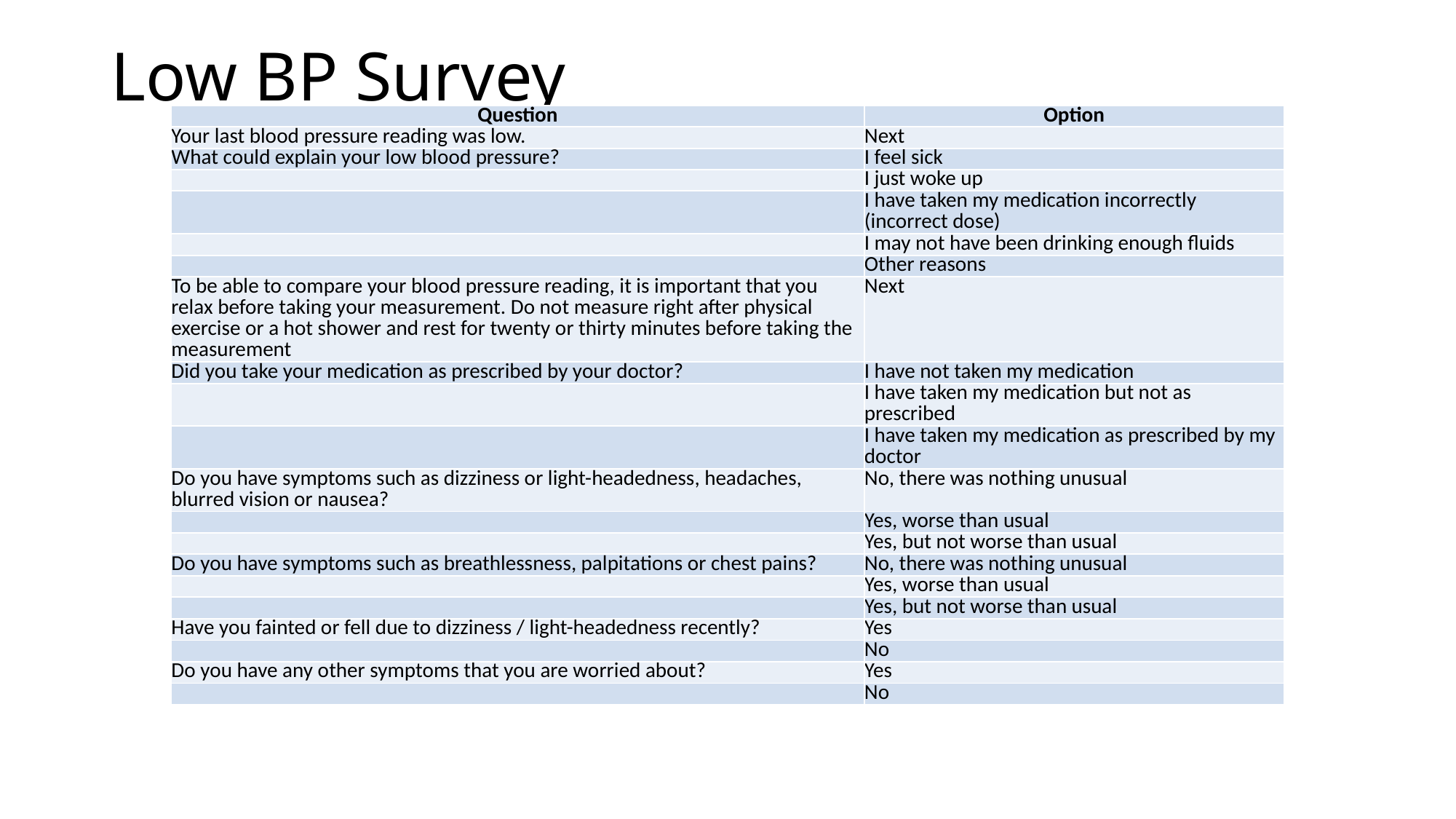

# Low BP Survey
| Question | Option |
| --- | --- |
| Your last blood pressure reading was low. | Next |
| What could explain your low blood pressure? | I feel sick |
| | I just woke up |
| | I have taken my medication incorrectly (incorrect dose) |
| | I may not have been drinking enough fluids |
| | Other reasons |
| To be able to compare your blood pressure reading, it is important that you relax before taking your measurement. Do not measure right after physical exercise or a hot shower and rest for twenty or thirty minutes before taking the measurement | Next |
| Did you take your medication as prescribed by your doctor? | I have not taken my medication |
| | I have taken my medication but not as prescribed |
| | I have taken my medication as prescribed by my doctor |
| Do you have symptoms such as dizziness or light-headedness, headaches, blurred vision or nausea? | No, there was nothing unusual |
| | Yes, worse than usual |
| | Yes, but not worse than usual |
| Do you have symptoms such as breathlessness, palpitations or chest pains? | No, there was nothing unusual |
| | Yes, worse than usual |
| | Yes, but not worse than usual |
| Have you fainted or fell due to dizziness / light-headedness recently? | Yes |
| | No |
| Do you have any other symptoms that you are worried about? | Yes |
| | No |

## Slide 14
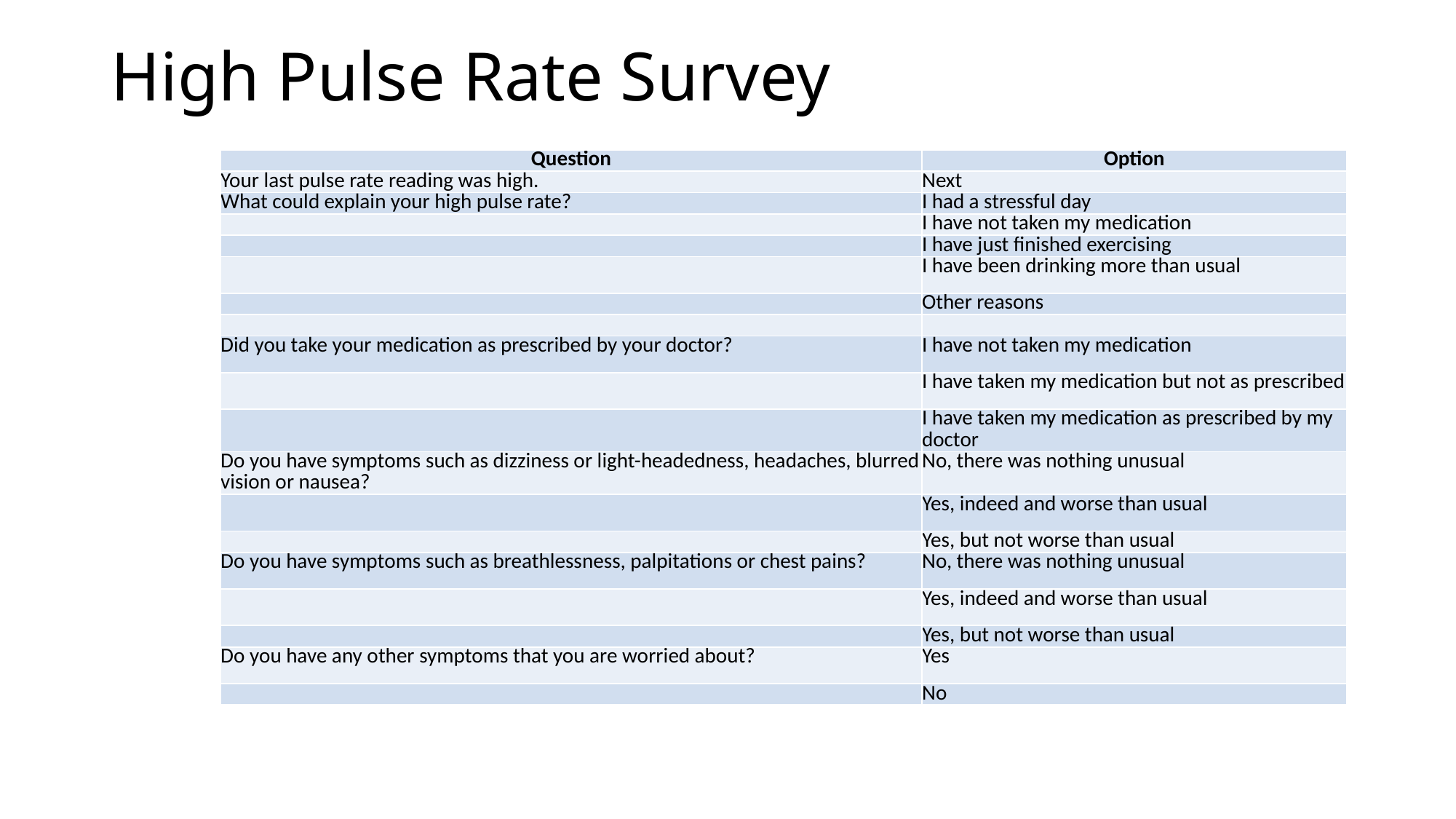

# High Pulse Rate Survey
| Question | Option |
| --- | --- |
| Your last pulse rate reading was high. | Next |
| What could explain your high pulse rate? | I had a stressful day |
| | I have not taken my medication |
| | I have just finished exercising |
| | I have been drinking more than usual |
| | Other reasons |
| | |
| Did you take your medication as prescribed by your doctor? | I have not taken my medication |
| | I have taken my medication but not as prescribed |
| | I have taken my medication as prescribed by my doctor |
| Do you have symptoms such as dizziness or light-headedness, headaches, blurred vision or nausea? | No, there was nothing unusual |
| | Yes, indeed and worse than usual |
| | Yes, but not worse than usual |
| Do you have symptoms such as breathlessness, palpitations or chest pains? | No, there was nothing unusual |
| | Yes, indeed and worse than usual |
| | Yes, but not worse than usual |
| Do you have any other symptoms that you are worried about? | Yes |
| | No |

## Slide 15
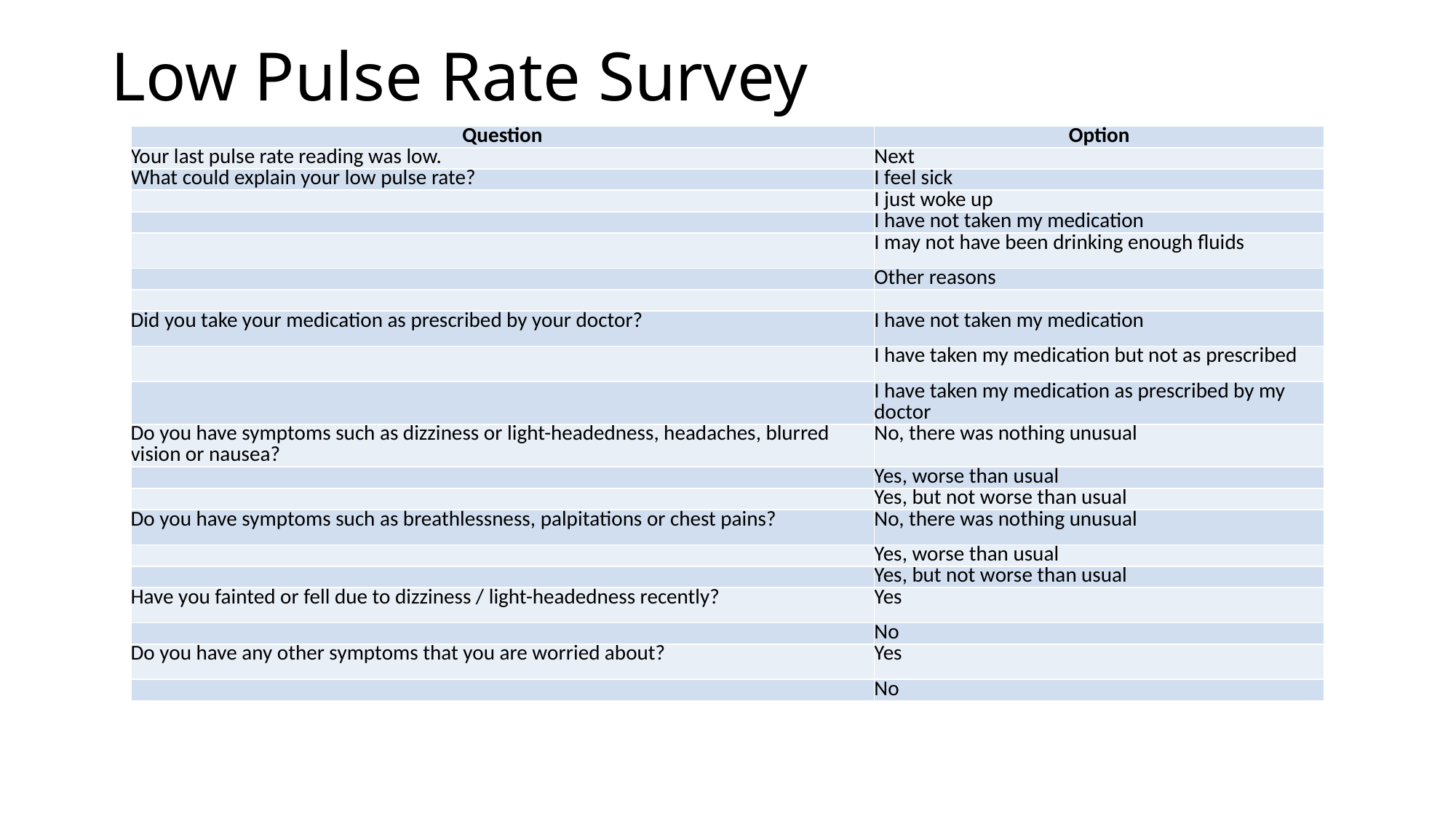

# Low Pulse Rate Survey
| Question | Option |
| --- | --- |
| Your last pulse rate reading was low. | Next |
| What could explain your low pulse rate? | I feel sick |
| | I just woke up |
| | I have not taken my medication |
| | I may not have been drinking enough fluids |
| | Other reasons |
| | |
| Did you take your medication as prescribed by your doctor? | I have not taken my medication |
| | I have taken my medication but not as prescribed |
| | I have taken my medication as prescribed by my doctor |
| Do you have symptoms such as dizziness or light-headedness, headaches, blurred vision or nausea? | No, there was nothing unusual |
| | Yes, worse than usual |
| | Yes, but not worse than usual |
| Do you have symptoms such as breathlessness, palpitations or chest pains? | No, there was nothing unusual |
| | Yes, worse than usual |
| | Yes, but not worse than usual |
| Have you fainted or fell due to dizziness / light-headedness recently? | Yes |
| | No |
| Do you have any other symptoms that you are worried about? | Yes |
| | No |

## Slide 16
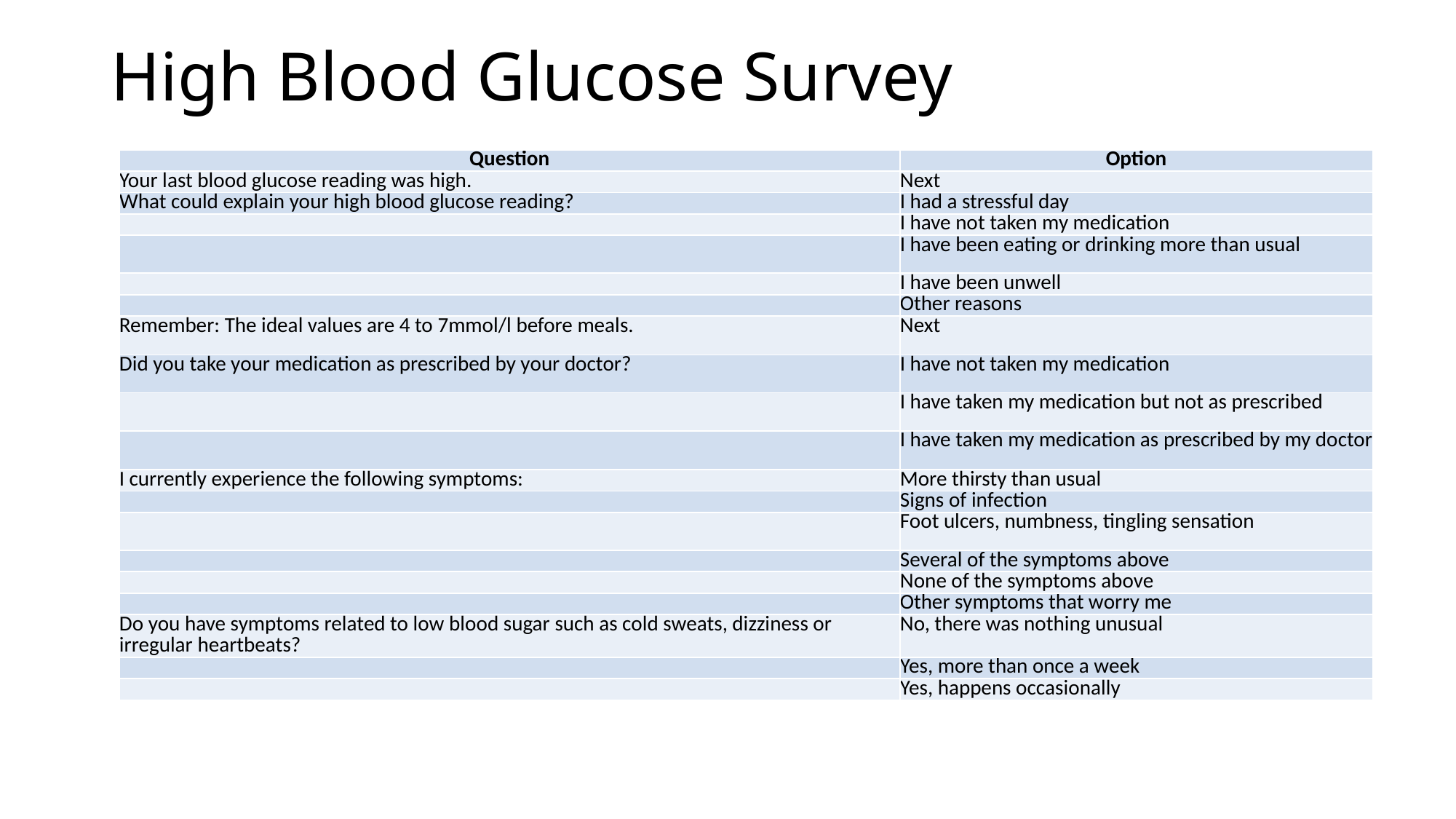

# High Blood Glucose Survey
| Question | Option |
| --- | --- |
| Your last blood glucose reading was high. | Next |
| What could explain your high blood glucose reading? | I had a stressful day |
| | I have not taken my medication |
| | I have been eating or drinking more than usual |
| | I have been unwell |
| | Other reasons |
| Remember: The ideal values are 4 to 7mmol/l before meals. | Next |
| Did you take your medication as prescribed by your doctor? | I have not taken my medication |
| | I have taken my medication but not as prescribed |
| | I have taken my medication as prescribed by my doctor |
| I currently experience the following symptoms: | More thirsty than usual |
| | Signs of infection |
| | Foot ulcers, numbness, tingling sensation |
| | Several of the symptoms above |
| | None of the symptoms above |
| | Other symptoms that worry me |
| Do you have symptoms related to low blood sugar such as cold sweats, dizziness or irregular heartbeats? | No, there was nothing unusual |
| | Yes, more than once a week |
| | Yes, happens occasionally |

## Slide 17
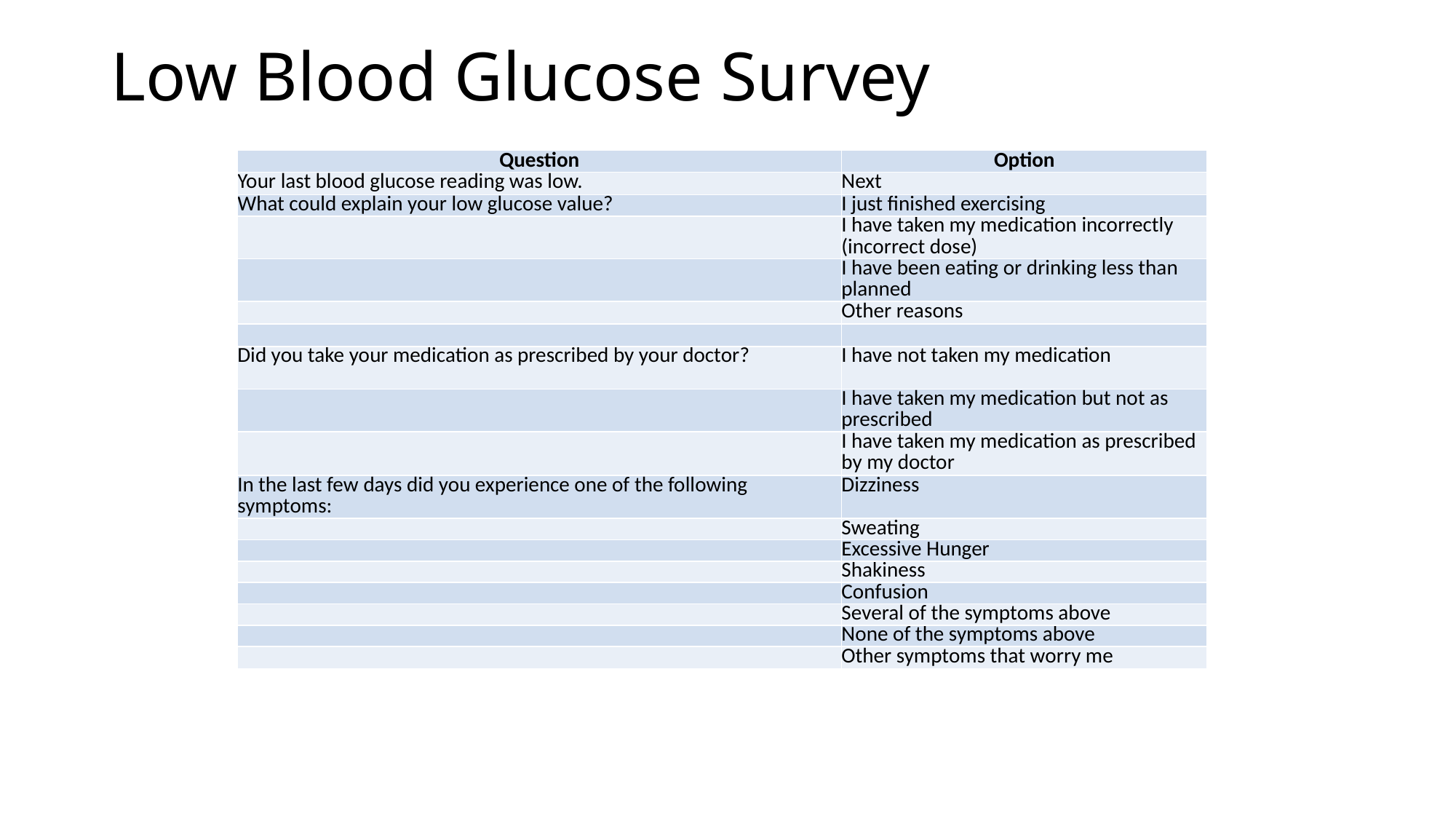

# Low Blood Glucose Survey
| Question | Option |
| --- | --- |
| Your last blood glucose reading was low. | Next |
| What could explain your low glucose value? | I just finished exercising |
| | I have taken my medication incorrectly (incorrect dose) |
| | I have been eating or drinking less than planned |
| | Other reasons |
| | |
| Did you take your medication as prescribed by your doctor? | I have not taken my medication |
| | I have taken my medication but not as prescribed |
| | I have taken my medication as prescribed by my doctor |
| In the last few days did you experience one of the following symptoms: | Dizziness |
| | Sweating |
| | Excessive Hunger |
| | Shakiness |
| | Confusion |
| | Several of the symptoms above |
| | None of the symptoms above |
| | Other symptoms that worry me |

## Slide 18
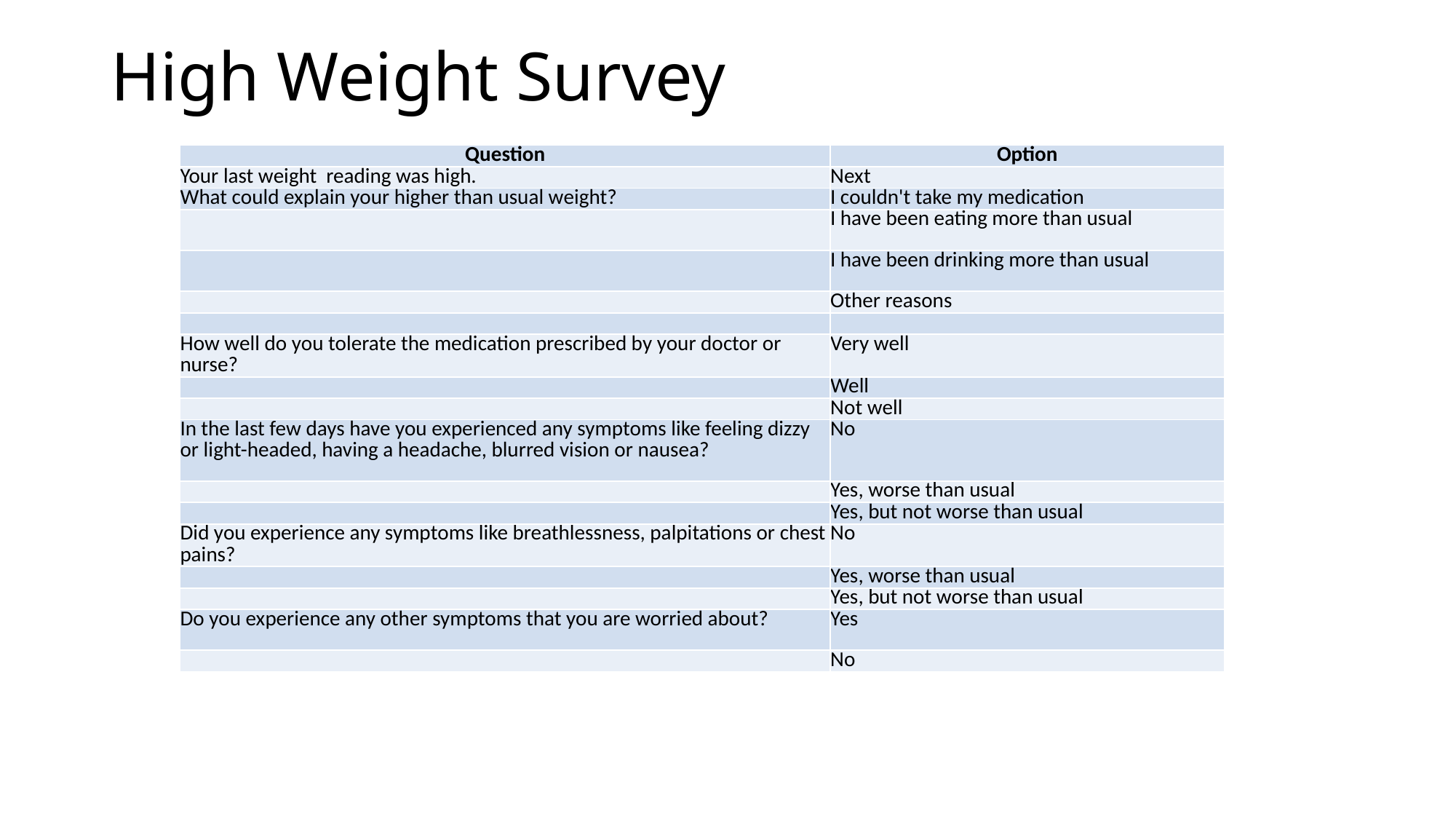

# High Weight Survey
| Question | Option |
| --- | --- |
| Your last weight reading was high. | Next |
| What could explain your higher than usual weight? | I couldn't take my medication |
| | I have been eating more than usual |
| | I have been drinking more than usual |
| | Other reasons |
| | |
| How well do you tolerate the medication prescribed by your doctor or nurse? | Very well |
| | Well |
| | Not well |
| In the last few days have you experienced any symptoms like feeling dizzy or light-headed, having a headache, blurred vision or nausea? | No |
| | Yes, worse than usual |
| | Yes, but not worse than usual |
| Did you experience any symptoms like breathlessness, palpitations or chest pains? | No |
| | Yes, worse than usual |
| | Yes, but not worse than usual |
| Do you experience any other symptoms that you are worried about? | Yes |
| | No |

## Slide 19
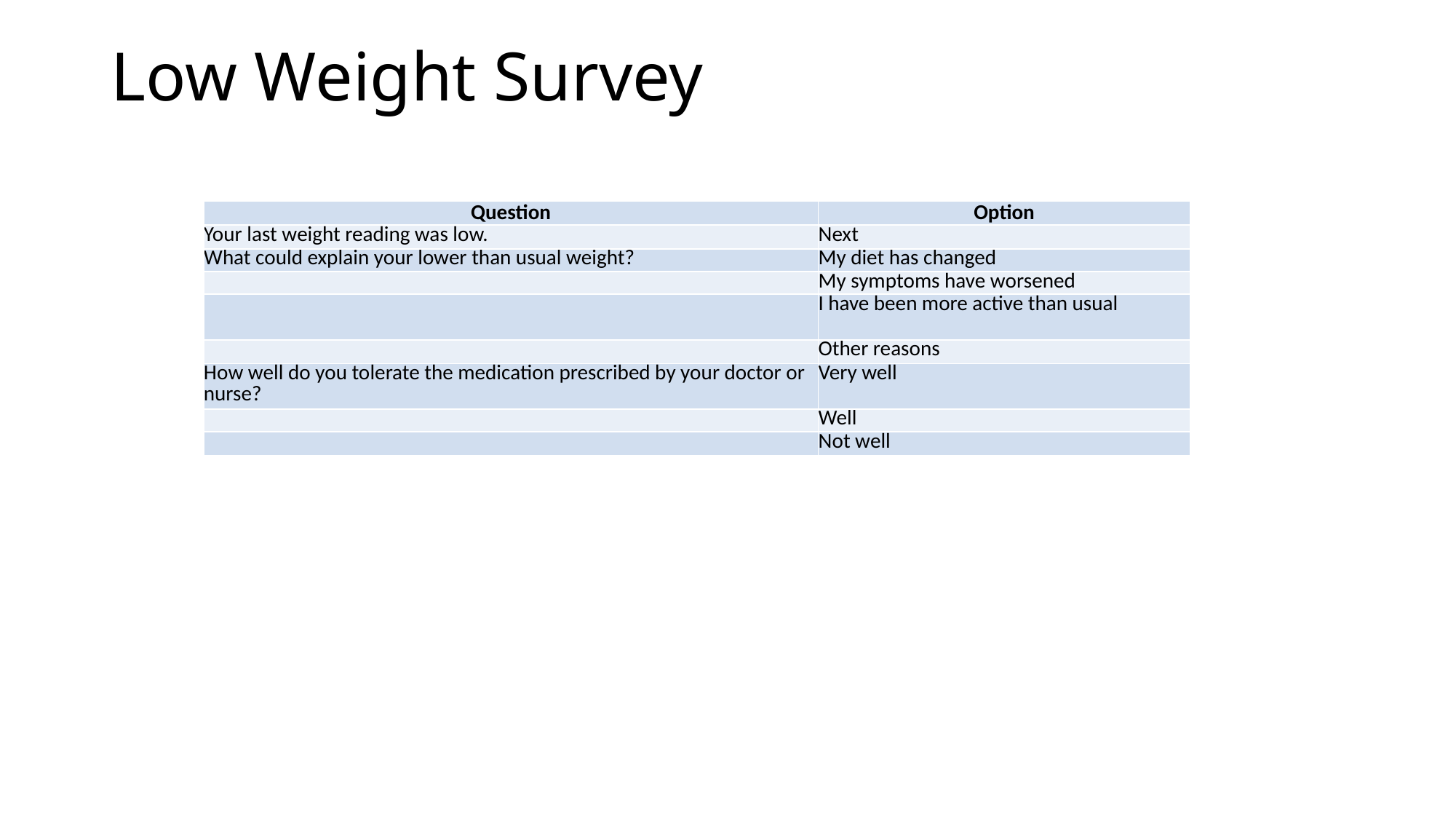

# Low Weight Survey
| Question | Option |
| --- | --- |
| Your last weight reading was low. | Next |
| What could explain your lower than usual weight? | My diet has changed |
| | My symptoms have worsened |
| | I have been more active than usual |
| | Other reasons |
| How well do you tolerate the medication prescribed by your doctor or nurse? | Very well |
| | Well |
| | Not well |

## Slide 20
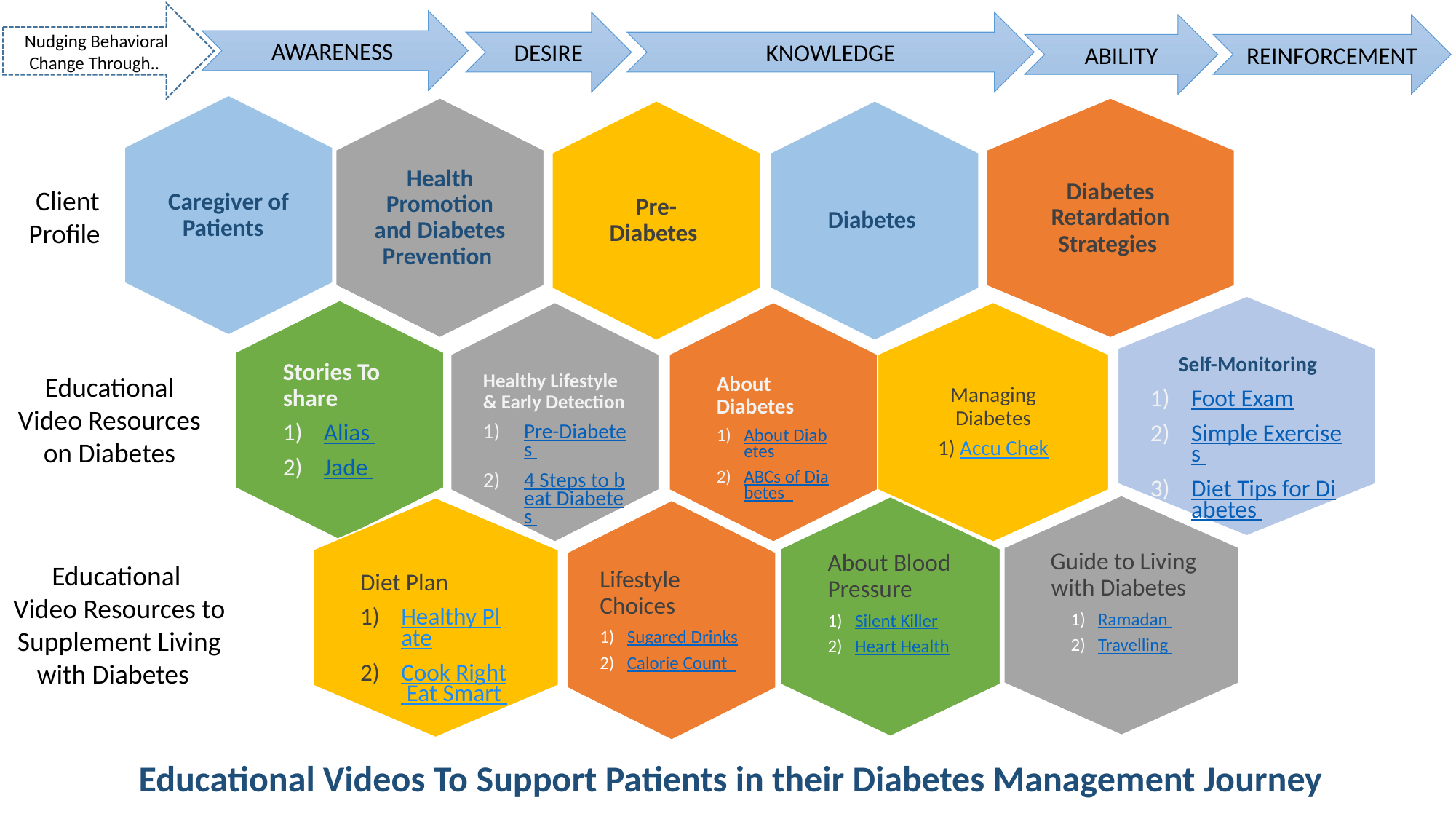

Nudging Behavioral Change Through..
AWARENESS
DESIRE
KNOWLEDGE
ABILITY
REINFORCEMENT
Caregiver of Patients
Health Promotion and Diabetes Prevention
Diabetes Retardation Strategies
Pre-Diabetes
Diabetes
Client Profile
 Self-Monitoring
Foot Exam
Simple Exercises
Diet Tips for Diabetes
Stories To share
Alias
Jade
Healthy Lifestyle & Early Detection
Pre-Diabetes
4 Steps to beat Diabetes
About Diabetes
About Diabetes
ABCs of Diabetes
Managing Diabetes
1) Accu Chek
Educational Video Resources on Diabetes
 Guide to Living with Diabetes
Ramadan
Travelling
About Blood Pressure
Silent Killer
Heart Health
Diet Plan
Healthy Plate
Cook Right Eat Smart
Lifestyle Choices
Sugared Drinks
Calorie Count
Educational
Video Resources to Supplement Living with Diabetes
Educational Videos To Support Patients in their Diabetes Management Journey

## Slide 21
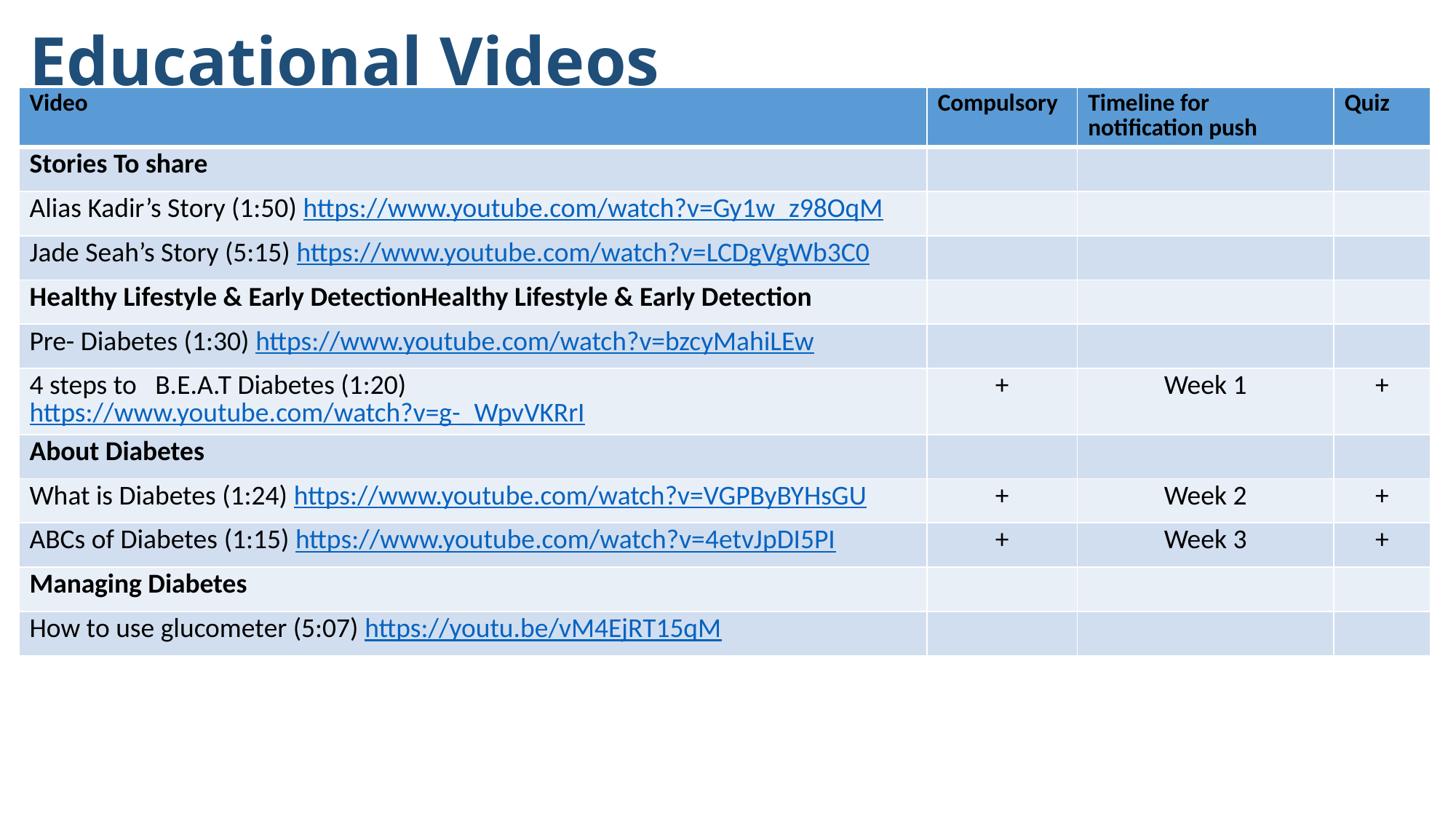

# Educational Videos
| Video | Compulsory | Timeline for notification push | Quiz |
| --- | --- | --- | --- |
| Stories To share | | | |
| Alias Kadir’s Story (1:50) https://www.youtube.com/watch?v=Gy1w\_z98OqM | | | |
| Jade Seah’s Story (5:15) https://www.youtube.com/watch?v=LCDgVgWb3C0 | | | |
| Healthy Lifestyle & Early DetectionHealthy Lifestyle & Early Detection | | | |
| Pre- Diabetes (1:30) https://www.youtube.com/watch?v=bzcyMahiLEw | | | |
| 4 steps to B.E.A.T Diabetes (1:20) https://www.youtube.com/watch?v=g-\_WpvVKRrI | + | Week 1 | + |
| About Diabetes | | | |
| What is Diabetes (1:24) https://www.youtube.com/watch?v=VGPByBYHsGU | + | Week 2 | + |
| ABCs of Diabetes (1:15) https://www.youtube.com/watch?v=4etvJpDI5PI | + | Week 3 | + |
| Managing Diabetes | | | |
| How to use glucometer (5:07) https://youtu.be/vM4EjRT15qM | | | |

## Slide 22
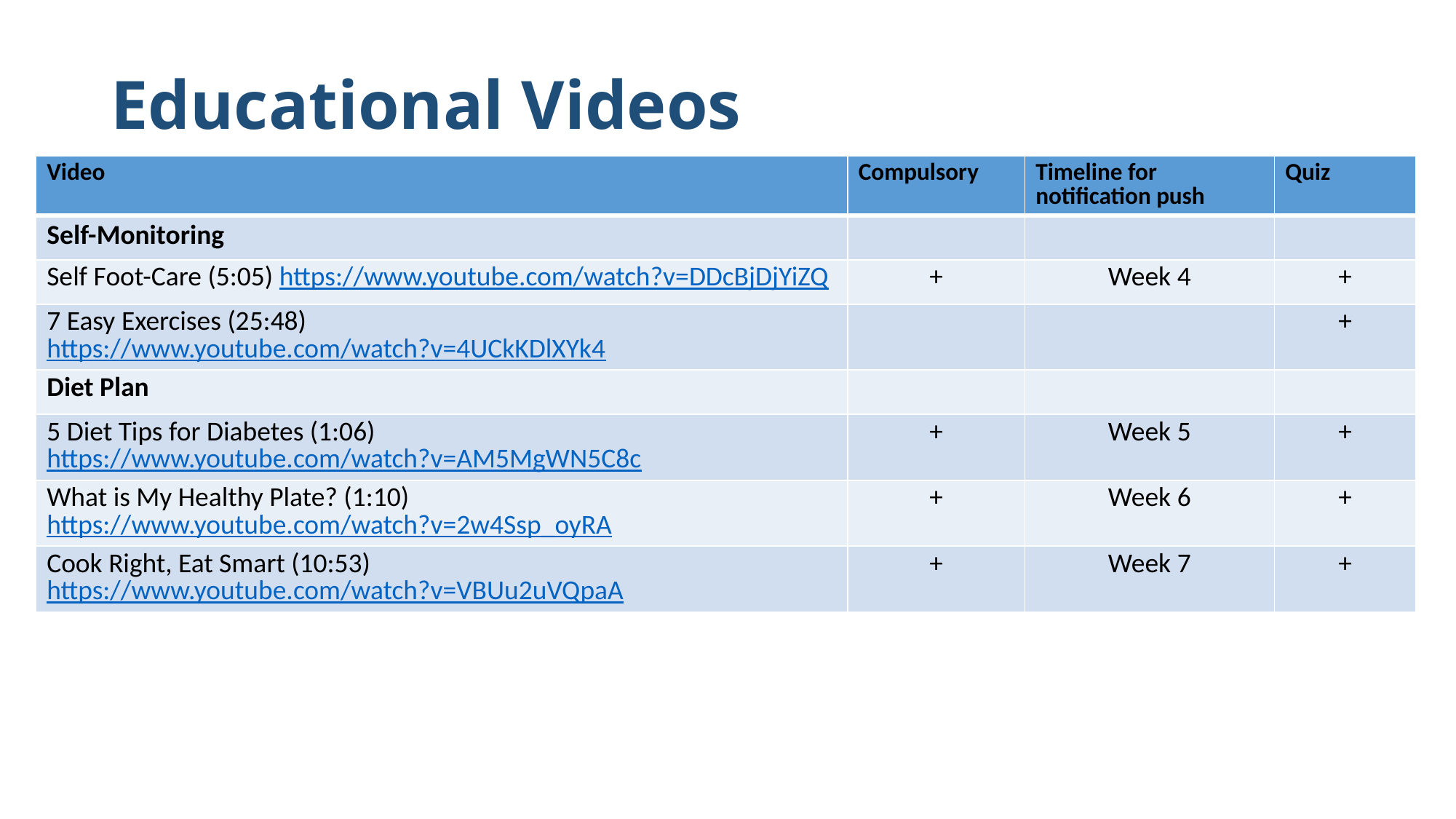

# Educational Videos
| Video | Compulsory | Timeline for notification push | Quiz |
| --- | --- | --- | --- |
| Self-Monitoring | | | |
| Self Foot-Care (5:05) https://www.youtube.com/watch?v=DDcBjDjYiZQ | + | Week 4 | + |
| 7 Easy Exercises (25:48) https://www.youtube.com/watch?v=4UCkKDlXYk4 | | | + |
| Diet Plan | | | |
| 5 Diet Tips for Diabetes (1:06) https://www.youtube.com/watch?v=AM5MgWN5C8c | + | Week 5 | + |
| What is My Healthy Plate? (1:10) https://www.youtube.com/watch?v=2w4Ssp\_oyRA | + | Week 6 | + |
| Cook Right, Eat Smart (10:53) https://www.youtube.com/watch?v=VBUu2uVQpaA | + | Week 7 | + |

## Slide 23
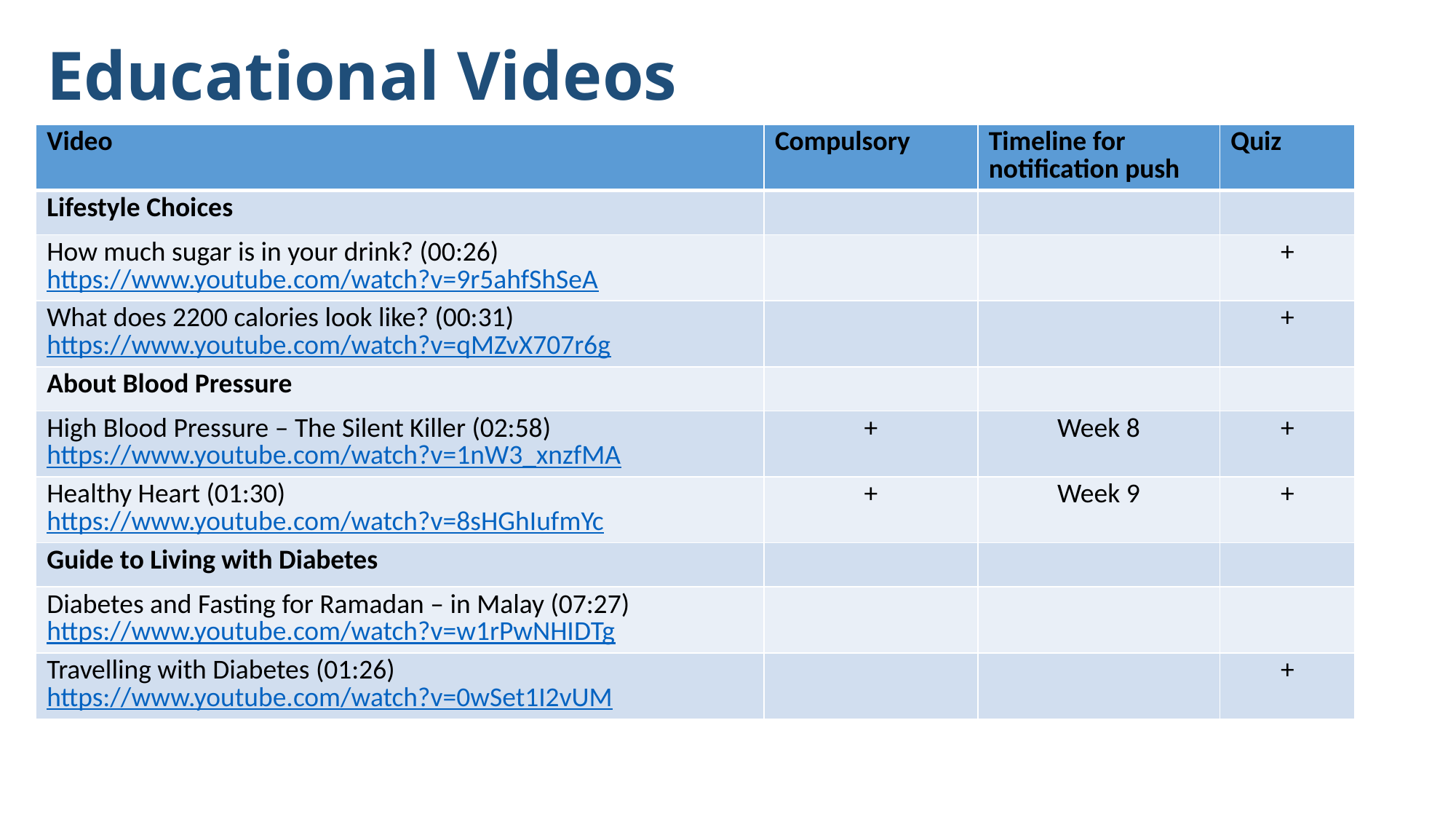

# Educational Videos
| Video | Compulsory | Timeline for notification push | Quiz |
| --- | --- | --- | --- |
| Lifestyle Choices | | | |
| How much sugar is in your drink? (00:26) https://www.youtube.com/watch?v=9r5ahfShSeA | | | + |
| What does 2200 calories look like? (00:31) https://www.youtube.com/watch?v=qMZvX707r6g | | | + |
| About Blood Pressure | | | |
| High Blood Pressure – The Silent Killer (02:58) https://www.youtube.com/watch?v=1nW3\_xnzfMA | + | Week 8 | + |
| Healthy Heart (01:30)https://www.youtube.com/watch?v=8sHGhIufmYc | + | Week 9 | + |
| Guide to Living with Diabetes | | | |
| Diabetes and Fasting for Ramadan – in Malay (07:27) https://www.youtube.com/watch?v=w1rPwNHIDTg | | | |
| Travelling with Diabetes (01:26) https://www.youtube.com/watch?v=0wSet1I2vUM | | | + |

## Slide 24
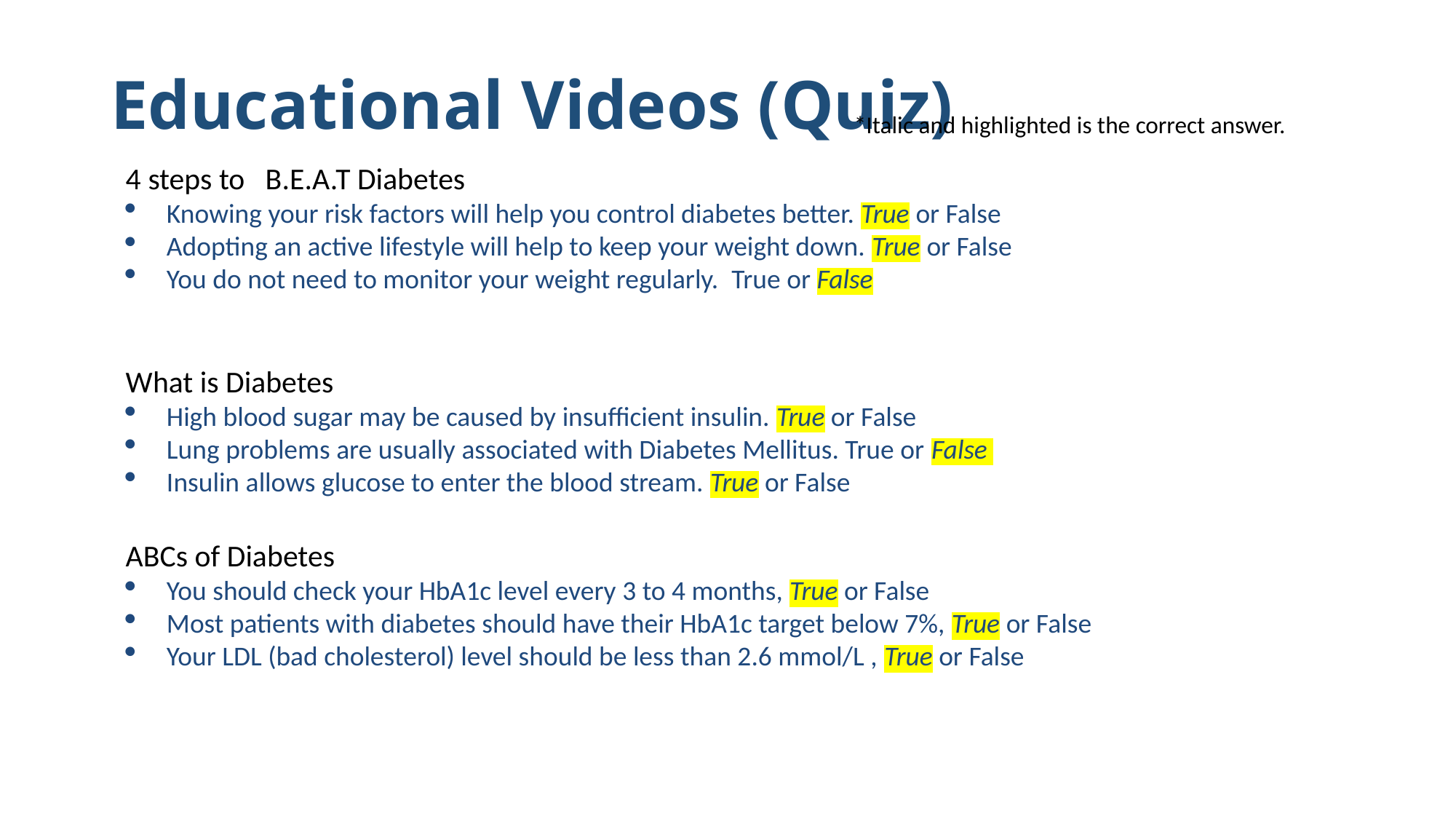

# Educational Videos (Quiz)
*Italic and highlighted is the correct answer.
4 steps to B.E.A.T Diabetes
Knowing your risk factors will help you control diabetes better. True or False
Adopting an active lifestyle will help to keep your weight down. True or False
You do not need to monitor your weight regularly.  True or False
What is Diabetes
High blood sugar may be caused by insufficient insulin. True or False
Lung problems are usually associated with Diabetes Mellitus. True or False
Insulin allows glucose to enter the blood stream. True or False
ABCs of Diabetes
You should check your HbA1c level every 3 to 4 months, True or False
Most patients with diabetes should have their HbA1c target below 7%, True or False
Your LDL (bad cholesterol) level should be less than 2.6 mmol/L , True or False

## Slide 25
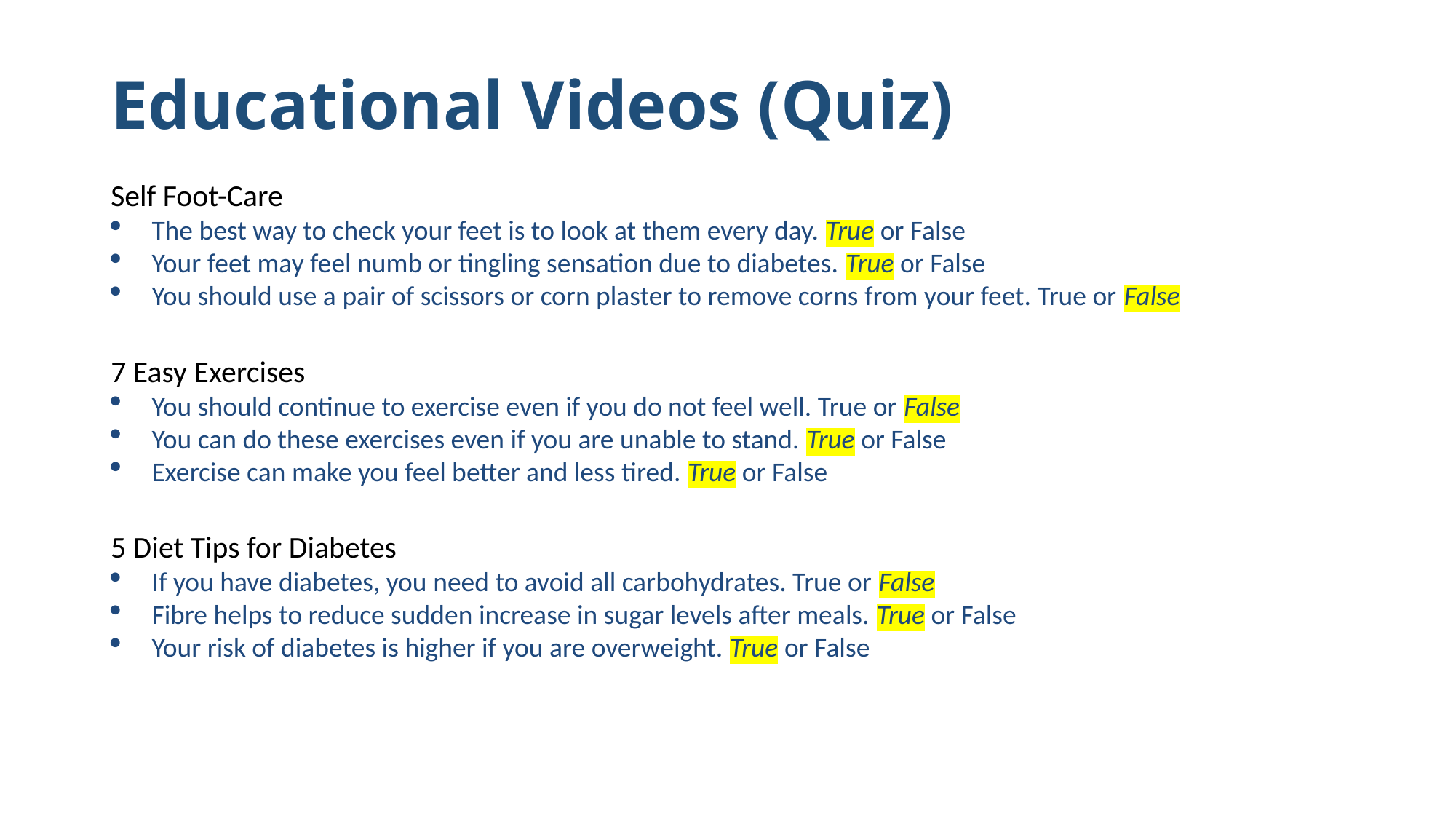

# Educational Videos (Quiz)
Self Foot-Care
The best way to check your feet is to look at them every day. True or False
Your feet may feel numb or tingling sensation due to diabetes. True or False
You should use a pair of scissors or corn plaster to remove corns from your feet. True or False
7 Easy Exercises
You should continue to exercise even if you do not feel well. True or False
You can do these exercises even if you are unable to stand. True or False
Exercise can make you feel better and less tired. True or False
5 Diet Tips for Diabetes
If you have diabetes, you need to avoid all carbohydrates. True or False
Fibre helps to reduce sudden increase in sugar levels after meals. True or False
Your risk of diabetes is higher if you are overweight. True or False

## Slide 26
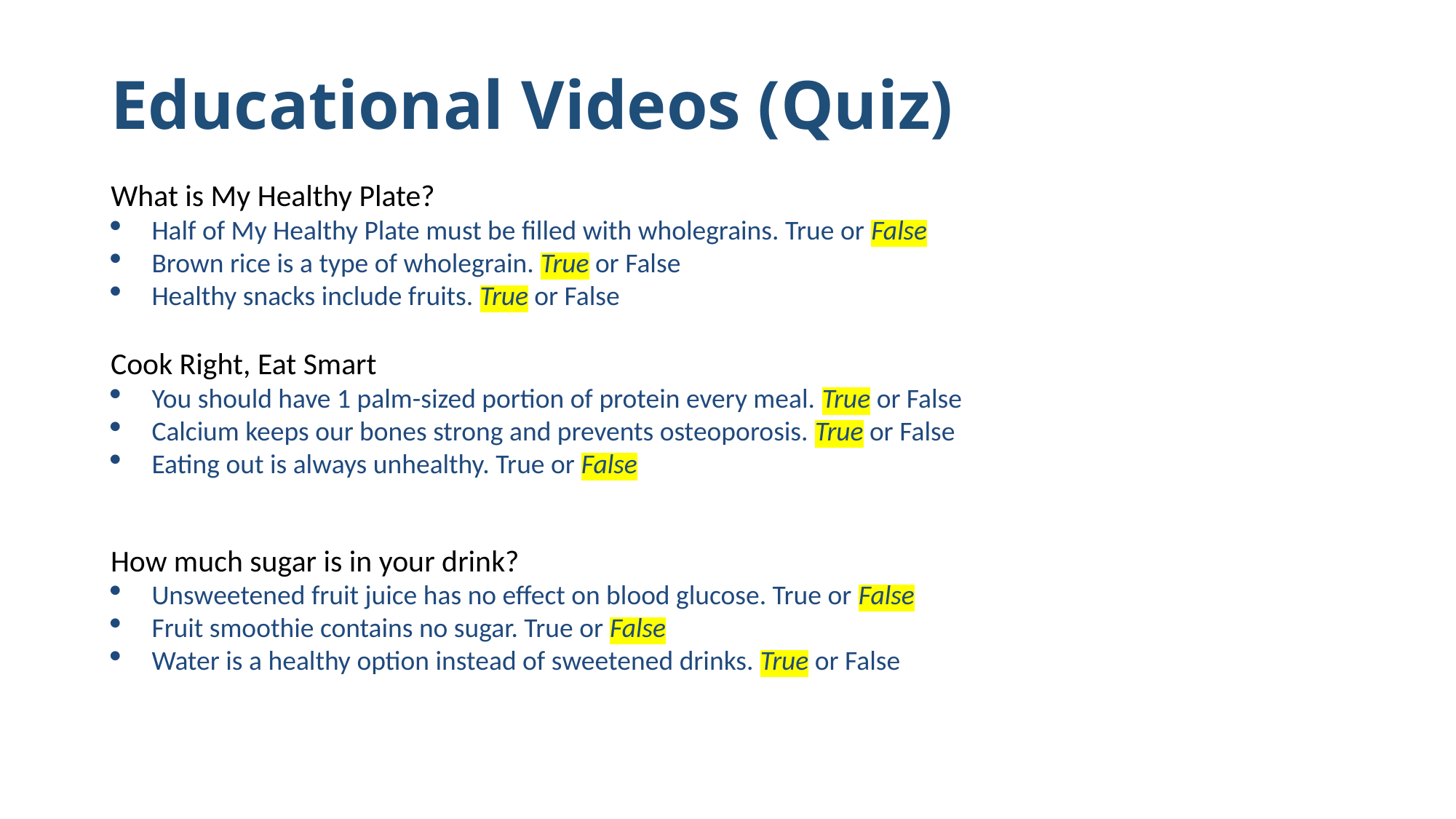

# Educational Videos (Quiz)
What is My Healthy Plate?
Half of My Healthy Plate must be filled with wholegrains. True or False
Brown rice is a type of wholegrain. True or False
Healthy snacks include fruits. True or False
Cook Right, Eat Smart
You should have 1 palm-sized portion of protein every meal. True or False
Calcium keeps our bones strong and prevents osteoporosis. True or False
Eating out is always unhealthy. True or False
How much sugar is in your drink?
Unsweetened fruit juice has no effect on blood glucose. True or False
Fruit smoothie contains no sugar. True or False
Water is a healthy option instead of sweetened drinks. True or False

## Slide 27
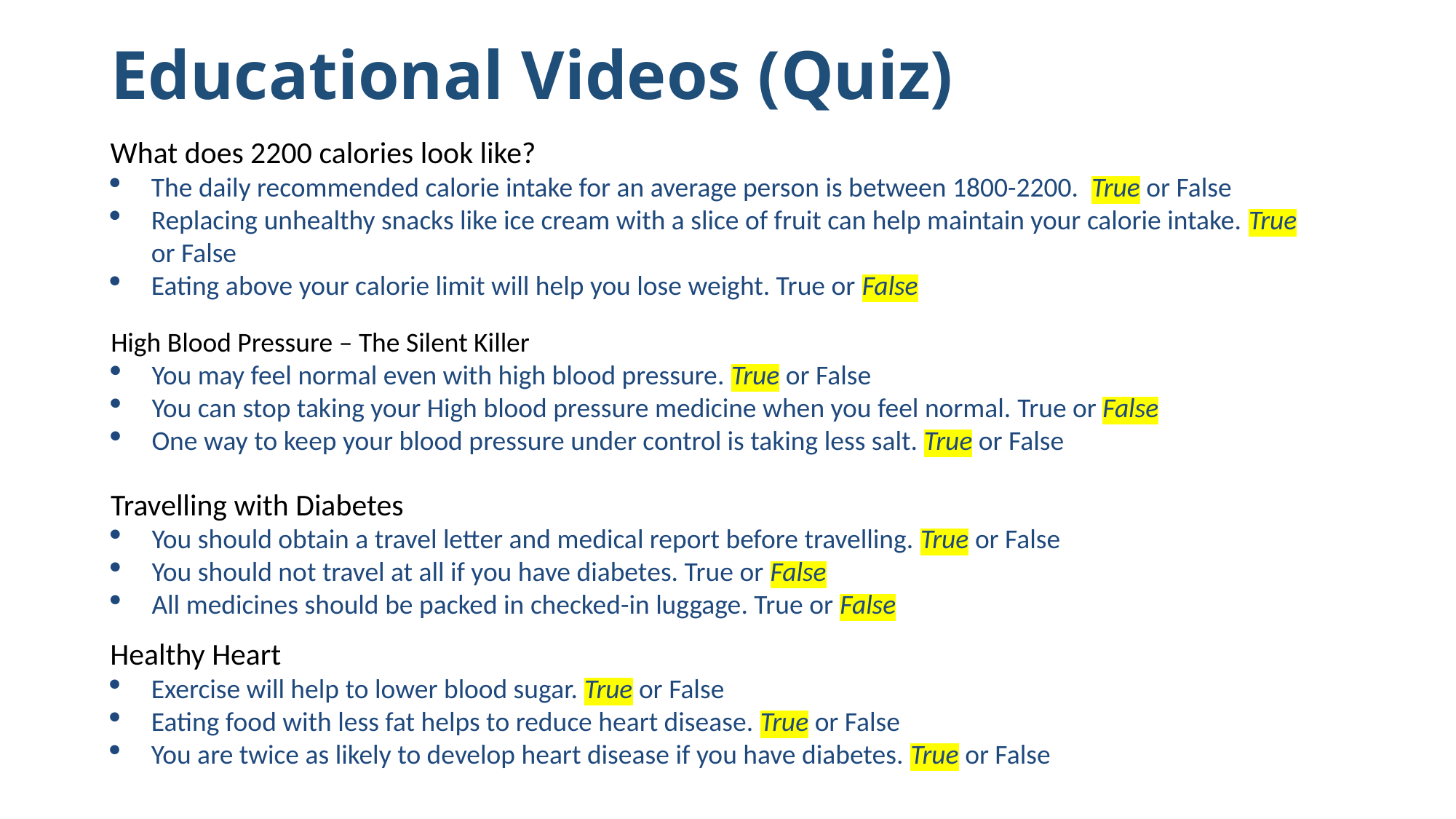

# Educational Videos (Quiz)
What does 2200 calories look like?
The daily recommended calorie intake for an average person is between 1800-2200.  True or False
Replacing unhealthy snacks like ice cream with a slice of fruit can help maintain your calorie intake. True or False
Eating above your calorie limit will help you lose weight. True or False
High Blood Pressure – The Silent Killer
You may feel normal even with high blood pressure. True or False
You can stop taking your High blood pressure medicine when you feel normal. True or False
One way to keep your blood pressure under control is taking less salt. True or False
Travelling with Diabetes
You should obtain a travel letter and medical report before travelling. True or False
You should not travel at all if you have diabetes. True or False
All medicines should be packed in checked-in luggage. True or False
Healthy Heart
Exercise will help to lower blood sugar. True or False
Eating food with less fat helps to reduce heart disease. True or False
You are twice as likely to develop heart disease if you have diabetes. True or False

## Slide 28
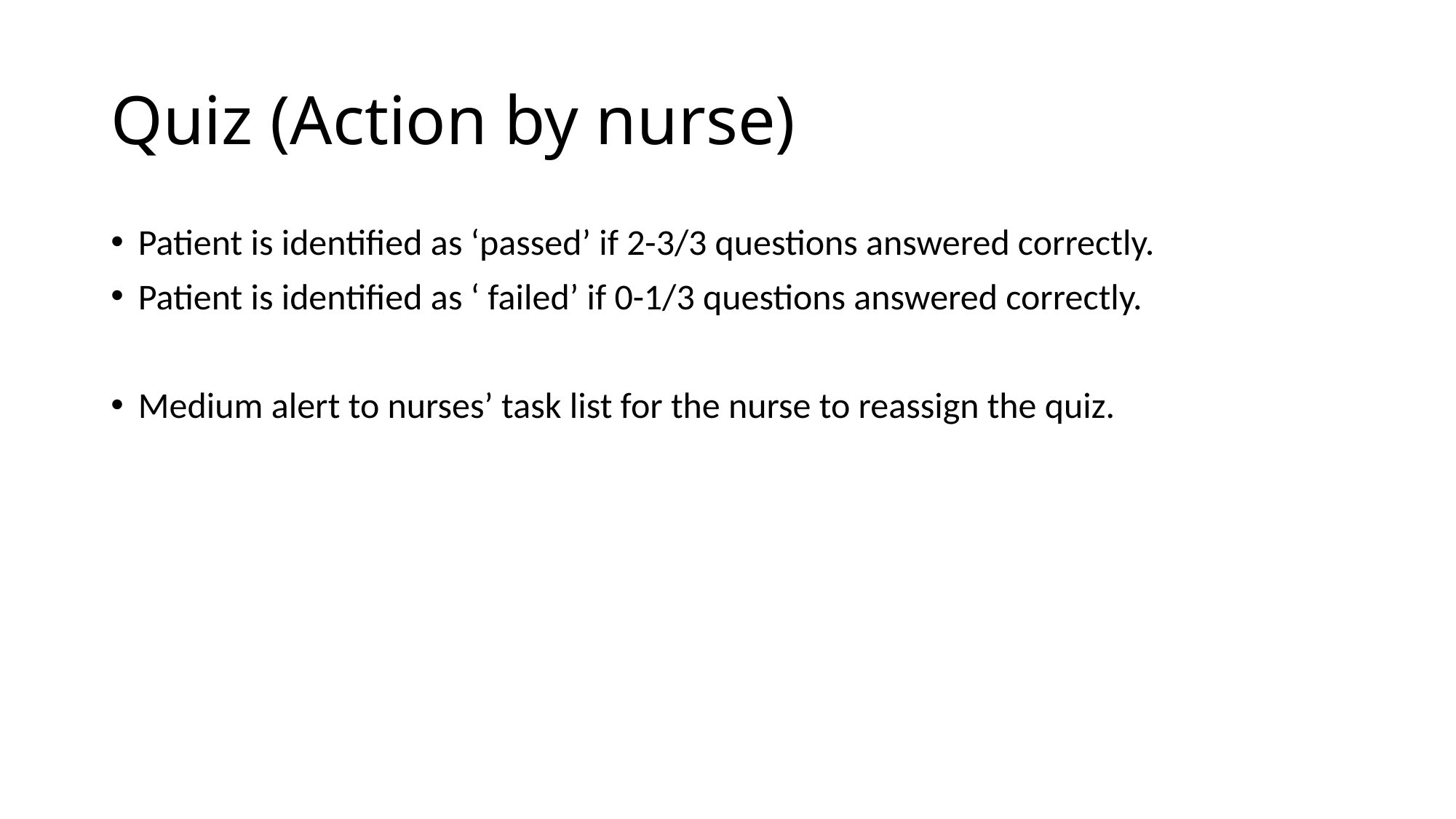

# Quiz (Action by nurse)
Patient is identified as ‘passed’ if 2-3/3 questions answered correctly.
Patient is identified as ‘ failed’ if 0-1/3 questions answered correctly.
Medium alert to nurses’ task list for the nurse to reassign the quiz.
