# Supplementary material for: Evaluating the effectiveness and utility of a novel culturally-adapted telemonitoring system in improving the glycaemic control of Asians with type-2 diabetes mellitus: a mixed method study protocol
Source: Trials. 2021 Apr 26;22:305. doi: 10.1186/s13063-021-05240-6 (PMC8072297; doi:10.1186/s13063-021-05240-6)
Supplement: Supplementary file 3 — Additional file 3. Topic guide for the qualitative study. [file 13063_2021_5240_MOESM3_ESM.docx]

| **INTERVIEW GUIDE (PATIENTS/CAREGIVERS) – Intervention Group** |
| --- |
| **Opening Questions**   - Can you tell me what it is like for you to have high blood glucose? - Do you have high blood pressure?   - Can you tell me what it is like to have different health problems, like high blood pressure and high blood glucose? - Can you tell me why you signed up for this telehealth Trial? |
| **Experience of participating in Telehealth Pilot Trial**   - I am interested in hearing about your experience of monitoring your blood pressure/glucose/weight using telemonitoring equipment, can you tell me all about it?   - PROBE: differences in monitoring blood pressure versus blood glucose versus weight; different ease of monitoring of different parameters etc. - Can you share about what you liked about trial? - Can you tell me about anything you did not like? - What would be some of the advantages of telemonitoring of blood pressure/glucose/weight? - Can you tell me about some disadvantages? - How do you feel about your blood pressure/glucose/weight now? - What would be some of the advantages of tele treatment (consultation) of blood pressure/glucose/weight? - Can you tell me about some disadvantages? |
| Do you think you would choose to keep using the telemonitoring equipment and self-monitor your blood pressure/glucose/weight if it was still available after 6 months?   - Why? - Why not? |
| Do you think you would choose to keep using the tele treatment (consultation and medication adjustment) and self-monitor your blood pressure/glucose/weight if it was still available after 6 months?   - Why - Why not? |
| Would you recommend this telehealth pilot of blood pressure/glucose/weight to other people with high blood pressure/glucose/weight?  Do you have any suggestions for improvement? Please elaborate. |
| **Self-Monitoring Experience**   - Can you tell me what it was like to learn to use different tele-monitoring devices at home?   - PROBE: training for blood pressure monitoring device, blood glucose monitoring device and weighing scale; any differences - Can you share what is has been like to monitor your blood pressure/glucose/weight yourself? - How did it make you feel? - Can you tell me about any concerns that you might have about monitoring your blood pressure/glucose/weight? - Can you share about experiencing any technical difficulties with using the devices?   - If so, PROBE: what did you think of the support received to address technical difficulties? |
| **Adherence to monitoring schedule**  (You were asked to monitor/record your BP/blood glucose/weight daily over the study period of 6 months)   - What has it been like for you to try and monitor your BP/blood glucose/weight daily? - How did you feel about the instructions given regarding this? - Were there any difficulties in following the instructions and monitoring BP/blood glucose/weight?   - PROBE: for compliance issues - Can you tell me about any time you had to skip your BP monitoring? |
| **Teleconsultation Experience**   - Can you tell me if the teleconsultation appointments were convenient? - How easy was it for you get an appointment? - How did it make you feel? - Can you tell me about any concerns that you might have about teleconsultation? |
| **Feedback Messages/Phone call by case managers**   - What did you think about the feedback you received on your readings? |
| **Education Experience**   - How did you feel about the education content delivered in videos?   - PROBE: was it helpful or not, how have you used the information provided to help you manage your condition   - What did you like about it?   - What did you not like about it or would want to change? - How did you feel about the education received by the telehealth coach?   - PROBE: was it helpful or not, how have you used the information provided to help you manage your condition   - What did you like about it?   - What did you not like about it or would want to change? - Can you share any suggestions for improvement of this educational component of the intervention? |
| **Other healthy changes**   - Can you tell me about anything else that you tried to change in your lifestyle to manage your BP/blood glucose/weight? |
| Is there anything else that you will like to add that is not already covered?  Thank you so much for your time and participation. |

| **INTERVIEW GUIDE (PATIENTS/CAREGIVERS) – Control Group** |
| --- |
| **Opening Questions**   - Can you tell me what it is like for you to have high blood glucose? - Do you have high blood pressure?   - Can you tell me what it is like to have different health problems, like high blood pressure and high blood glucose? - Can you tell me why you signed up for this telehealth trial? |
| **Experience of participating in Telehealth Pilot Trial**   - I am interested in hearing about your experience of participating in this telehealth pilot trial, can you tell me all about it? |
| **General Questions about BP/Blood Glucose/Weight**   - Can you talk me through what (if anything) you currently do to manage for high blood pressure/glucose/weight? - Can you tell me how are your physician or nurse at polyclinic or other polyclinic staff involved in helping you to manage your blood pressure/glucose/weight? - Can you share about what you liked about polyclinic visits (if any)? - Can you tell me about anything you did not like? - Can you tell me what it is like for you taking medications for your high blood pressure/glucose? - Can you tell me what you feel about your physician making changes to your medicine dosage? |
| **Self-Monitoring Experience**   - Do you monitor your blood pressure/glucose/weight at home?   - Were you monitoring your BP/glucose/weight before entering this telehealth pilot trial?   - How do you currently monitor your BP/glucose/weight at home? - Can you tell me what it was like to use BP/glucose/weight monitor at home? - What do you do with the information?   - Probe: keep track of hypertension/diabetes/weight, share with FP in next visit, modify your diet/exercise etc. - How did it make you feel? - Can you tell me about any concerns that you might have about monitoring your blood pressure/glucose/weight? |
| **Other healthy changes**   - Can you tell me about anything else that you tried to change in your lifestyle to manage your BP? |
| Is there anything else that you will like to add that is not already covered?  Thank you so much for your time and participation. |
